# Histone methyltransferase Smyd2 drives adipogenesis via regulating STAT3 phosphorylation

**DOI:** 10.1038/s41419-022-05321-7

**Published:** 2022-10-21

**Authors:** Haibi Su, Chen Meng, Jie Xu, Zhenghua Su, Chenxi Xiao, Di Yang

**Affiliations:** Pharmacophenomics Laboratory, Human Phenome Institute, Zhangjiang Fudan International Innovation Center, Fudan University, Shanghai, 201203 P. R. China

**Keywords:** Epigenetics, Obesity

## Abstract

Adipogenesis is a complex cascade involved with the preadipocytes differentiation towards mature adipocytes, accelerating the onset of obesity. Histone methyltransferase SET and MYND domain-containing protein 2 (Smyd2), is involved in a variety of cellular biological functions but the epigenetic regulation of Smyd2 in adipogenesis and adipocyte differentiation remains unclear. Both *Smyd2* siRNA and LLY-507, an inhibitor of Smyd2, were used to examine the effect of Smyd2 on adipogenesis and adipocyte differentiation in vitro. Smyd2 heterozygous knockout (*Smyd2*^+/−^) mice were also constructed to validate the relationship between Smyd2 and adipogenesis in vivo. We found that Smyd2 is abundant in white adipose tissue and closely correlated with adipocyte differentiation. Knockdown or inhibition of Smyd2 restrained adipocyte differentiation in vitro, which requires the phosphorylation of STAT3. In vivo functional validation, *Smyd2*^+/−^ mice exert significant fat loss but not susceptible to HFD-induced obesity. Taken together, our findings revealed that Smyd2 is a novel regulator of adipocyte differentiation by regulating the phosphorylation of STAT3, which provides insights into the effects of epigenetic regulation in adipogenesis. Inhibition of Smyd2 might represent a viable strategy for anti-adipogenesis and maybe further alleviate obesity-related diseases in humans.

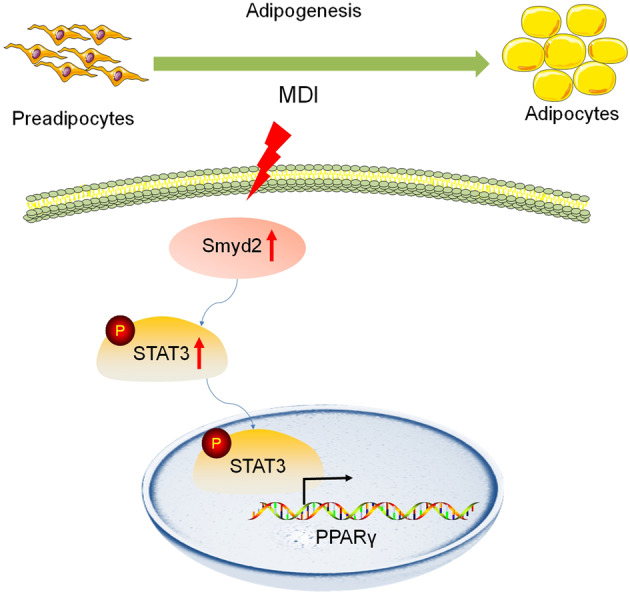

## Introduction

As of today, obesity has been recognized as the major burden and concern of worldwide public health and nearly 2 billion people worldwide are overweight or obese according to the World Health Organization [1, 2]. Although efforts have been made to develop anti-obesity drugs, unfortunately, little has been achieved [3]. Compelling evidence indicates that the rise in adipocyte hyperplasia and hypertrophy is correlated with an excessive accumulation of adipose tissue, which has become the research focus due to the prevalence of metabolic diseases [4, 5]. Therefore, investigating the novel targets and molecular mechanism of adipogenesis may provide potential therapeutic approaches to limit adipose expansion and further set a crucial basis for treatments of obesity and its associated comorbidities [6].

Numerous studies have revealed that adipogenesis is involved sequential activation of a complex network of key transcription factors during adipocyte differentiation, including the peroxisome proliferator‐activated receptor γ (PPARγ) and CCAAT/enhancer-binding protein (C/EBP) α, β, and δ, which act as the key master regulators [7]. In particular, PPARγ transcriptionally regulates the expressions of target genes associated with lipid storage and metabolism, such as fatty acid‐binding protein (FABP4) [8], accelerating the adipocyte differentiation and further establishing the mature adipocyte phenotypes. Furthermore, fatty acid synthase (FASN) and acetyl CoA carboxylase (ACC) are the principal enzymes of de novo adipogenesis and critical for adipocyte development and function.

Many studies have well-documented the transcriptional regulation of adipogenesis [9], but little is known about the mechanism of epigenetic regulation in adipogenesis, specifically histone modifications. Histone methylations mainly generate mono-, di-, or tri-methylation at the lysine residues at various lysine sites of H3 (H3K4, H3K9, H3K27, H3K36, and H3K79) or H4K20 by methyltransferases or demethylases, further resulting in either gene activation or repression [10]. Smyd2 is one of the SET and MYND-containing lysine methyltransferases (SMYD) family members and performs diverse functions through methylating both histones and non-histones [11]. The activity of SMYD2 is essential for the normal development and regulation of a range of pathophysiological processes in the organism. SMYD2 can exert different effects in different cells and organs through the regulation of different substrates. Since the abnormal expression or dysfunction of SMYD2 is often closely associated with a variety of diseases, SMYD2 is regarded as a promising candidate for the treatment of diseases, such as cardiovascular disease and cancer [12]. For instance, recent proteomic analysis has identified numerous substrate proteins of Smyd2, such as p53, RB et al. in several types of cancers [12, 13]. Besides, Smyd2 is reported to be dispensable for the development of mouse heart [14], and loss of Smyd2 causes the aberrant tail formation and developmental delay in zebrafish [15]. Furthermore, the methylation status of the Smyd2 promoter causes Smyd2 expression to be greatly downregulated in abdominal aortic aneurysm (AAA), according to genome-wide association studies (GWAS) [16]. Moreover, Smyd2 regulates autosomal dominant polycystic kidney disease by methylation and activation of STAT3 and p65 [17]. Recent studies have shown that overexpression of Smyd2 promotes aerobic glycolysis and reveals a novel link between Smyd2 and tumor metabolism [18]. Importantly, the protein levels of SMYD2 were reduced in diabetic nephropathy (DN) mice after Ranunculus ternatus Thunb (Ranunculaceae, RTT) extract treatment [19], suggesting that Smyd2 may play a role in metabolic diseases. However, the contribution of Smyd2 in adipogenesis and obesity remains unknown.

In the present study, we showed that Smyd2 is abundant in white adipose tissue and closely correlated with adipocyte differentiation. Knockdown of Smyd2 by siRNA or inhibition of Smyd2 by the potent and selective inhibitor LLY-507 restrained adipocyte differentiation in vitro, which required the phosphorylation of STAT3. Furthermore, *Smyd2*^+/−^ mice exert significant fat loss but are not susceptible to HFD-induced obesity in vivo. Our findings revealed that Smyd2 contributes to the process of adipocyte differentiation and inhibiting Smyd2 might represent a viable strategy for the therapy of obesity in humans.

## Results

### Smyd2 is abundant in white adipose tissue

**Fig. 1:**
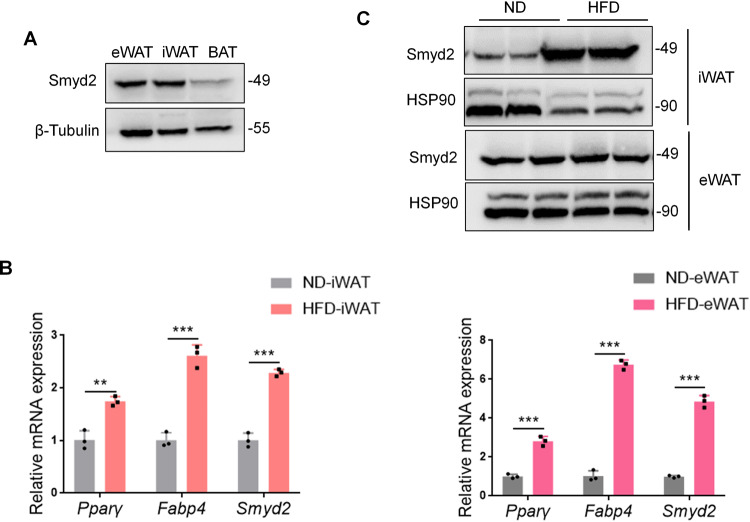
Smyd2 is abundant in white adipose tissue. **A** The expression profile of Smyd2 in adipose tissues. The inguinal white adipose tissue (iWAT), epididymal white adipose tissue (eWAT), and brown adipose tissue (BAT) from adult C57BL/6J mice were harvested and examined the protein expression of Smyd2 by western blot assay. *n* = 3 mice/groups. **B** The mRNA levels of *Smyd2* and the adipocyte marker genes (*Pparγ*, *Fabp4*) were detected in iWAT and eWAT of HFD-induced obese mice and ND-fed control mice. *n* = 3 mice/groups. **C** The protein expressions of Smyd2 were detected in iWAT and eWAT of HFD-induced obese mice and ND-fed control mice. *n* = 3 mice/groups. Data are presented as mean ± SD. **p < 0.01, ***p < 0.001.

To explore the expression profile of Smyd2 in adipose tissues, we harvested inguinal white adipose tissue (iWAT), epididymal white adipose tissue (eWAT), and brown adipose tissue (BAT) from adult C57BL/6J mice and found Smyd2 was highly expressed in WAT compared to BAT (Fig. 1A). Next, we determined the protein and mRNA levels of Smyd2 in iWAT and eWAT of high fat diet (HFD)-induced mice. As indicated in Fig. 1B, the mRNA level of Smyd2 was significantly upregulated in both iWAT and eWAT of HFD-induced obese mice than in normal diet (ND)-fed mice, accompanied by the upregulated pan-adipocyte marker genes such as *Pparγ* and *Fabp4*. While the protein expression of Smyd2 was only markedly increased in iWAT but not eWAT of HFD-induced obese mice (Fig. 1C). These data indicated that Smyd2 may play a key role during the adipogenesis of iWAT.

### Smyd2 is closely correlated with adipocyte differentiation in vitro

**Fig. 2:**
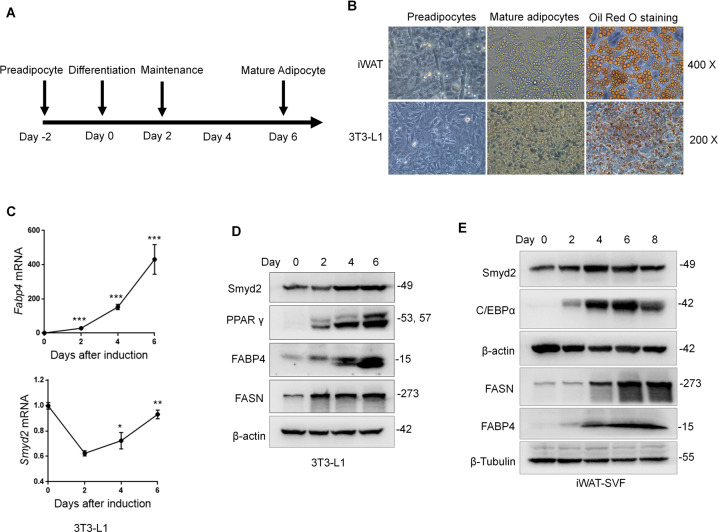
Smyd2 is closely correlated with adipocyte differentiation in vitro. **A** The schematic outline of experimental procedures for adipocyte differentiation. **B** 3T3-L1 cells and iWAT-SVF were differentiated into mature adipocytes, as detected by both the bright field of microscopy and the Oil Red O staining. Magnification ×400 and ×200. **C** The mRNA levels of *Smyd2* and the adipocyte marker gene (*Fabp4*) were examined during 3T3-L1 cell differentiation by RT-qPCR analysis. **D, E** The protein expressions of Smyd2 and adipocyte differentiation markers (PPARγ, FABP4, FASN, and CEBPα) during 3T3-L1 cells and iWAT-SVF differentiation were detected by immunoblot analysis. Data are presented as mean ± SD. *p < 0.05, **p < 0.01, ***p < 0.001.

To investigate whether Smyd2 plays a role in adipogenesis in vitro, we examined the changes of Smyd2 during 1-methyl-3-isobutylxanthine, dexamethasone, and insulin (MDI)-induced differentiation of 3T3-L1 cells, a well-recognized mouse preadipocyte cell line and stromal vascular fraction (SVF) cells from iWAT tissue (iWAT-SVF), respectively. Firstly, the in vitro adipocyte differentiation system was constructed successfully by detecting the fully staining of Oil Red O staining (Fig. 2A, B), as well as the upregulation of adipocyte differentiation markers, including PPARγ, FABP4, FASN, and C/EBPα (Fig. 2C–E). Delightfully, the immunoblot results showed a dramatic increase in Smyd2 expression during the differentiation of 3T3-L1 cells and iWAT-SVF (Fig. 2D, E). Furthermore, the RT-qPCR analyses revealed that the mRNA level of Smyd2 first downregulated after induction and then gradually upregulated during adipocyte differentiation (Fig. 2C), demonstrating that Smyd2 is closely correlated with the process of adipocyte differentiation.

### Knockdown of Smyd2 inhibits adipocyte differentiation in vitro

**Fig. 3:**
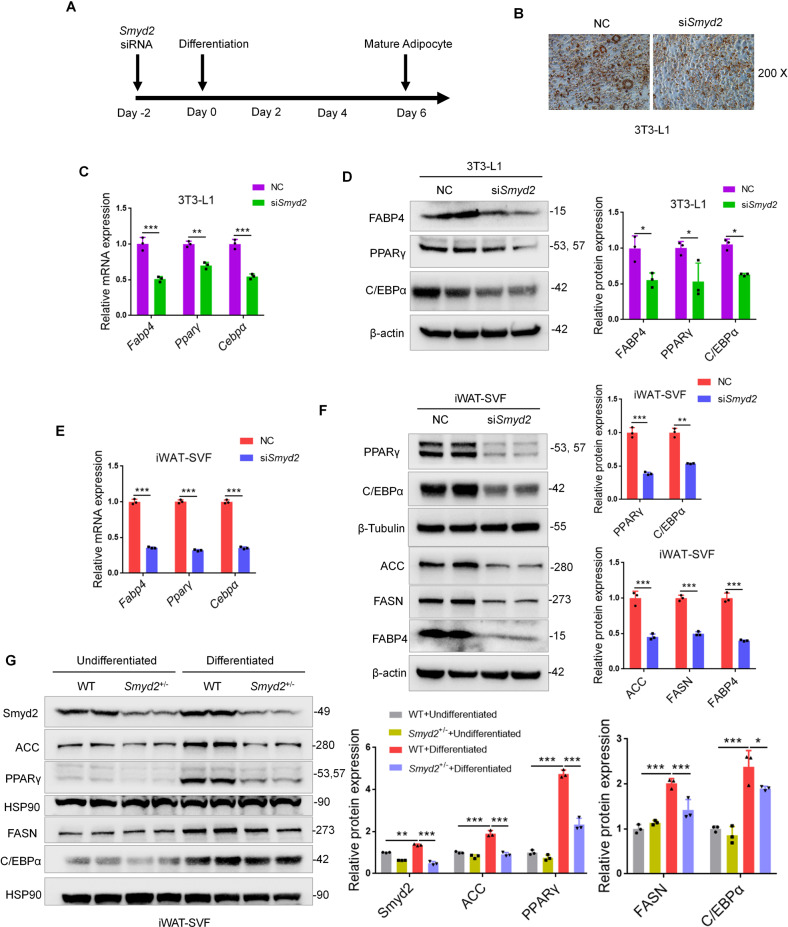
Knockdown of Smyd2 inhibits adipocyte differentiation in vitro. 3T3-L1 preadipocytes or iWAT-SVF were transfected with *Smyd2* siRNA or NC, followed by differentiation as described in the methods section. **A** The workflow for *Smyd2* siRNA transfection and adipocyte differentiation. **B** The Oil Red O staining of 3T3-L1 adipocytes with NC or Smyd2 siRNA transfection. Magnification × 200. **C** The mRNA levels of adipogenesis markers (*Pparγ*, *Cepbα*, and *Fabp4*) of 3T3-L1 adipocytes with NC or *Smyd2* siRNA transfection. **D** The protein expressions of adipogenesis markers (PPARγ, C/EBPα and FABP4) of 3T3-L1 adipocytes with NC or *Smyd2* siRNA transfection. **E** The mRNA levels of adipogenesis markers (*Pparγ*, *Cepbα*, and *Fabp4*) of iWAT-SVF adipocytes with NC or *Smyd2* siRNA transfection. **F** The protein expressions of adipogenesis markers (PPARγ, C/EBPα, FABP4), the rate-limiting enzyme in adipogenesis (ACC and FASN) of iWAT-SVF adipocytes with NC or *Smyd2* siRNA transfection. **G** The primary iWAT-SVF from WT and *Smyd2*^+/−^ mice were isolated and induced to undifferentiation or differentiation. Expression of Smyd2, adipogenesis markers (PPARγ, C/EBPα) and the rate-limiting enzymes in adipogenesis (ACC and FASN) were examined by western blot. Data are presented as mean ± SD. *p < 0.05, **p < 0.01, ***p < 0.001.

To further explore whether Smyd2 is necessary during adipocyte differentiation, 3T3-L1 preadipocytes or iWAT-SVF were transfected with *Smyd2* siRNA (si*Smyd2*) to knockdown Smyd2, then differentiated into mature adipocytes for 6 days (Fig. 3A). The knockdown efficiency of Smyd2 was verified in both mRNA and protein levels (Fig. S1A, B). Then we found that the adipocytes with *Smyd2* siRNA obtained fewer lipid droplets than the control cells, as detected by Oil Red O staining (Fig. 3B). Furthermore, the mRNA and protein levels of adipogenesis markers (PPARγ, C/EBPα, and FABP4) were significantly decreased at day 6 of 3T3-L1 cell differentiation after *Smyd2* siRNA treatment (Fig. 3C, D). In addition, similar patterns of changes were also observed in iWAT-SVF (Fig. 3E, F). Besides, the rate-limiting enzymes in adipogenesis (ACC and FASN) were also significantly decreased on day 6 of iWAT-SVF differentiation after *Smyd2* siRNA treatment (Fig. 3F).

Taking it a step further, we constructed Smyd2 heterozygous knockout (*Smyd2*^+/−^) mice and their littermate controls (WT) as described previously [20] and isolated the SVF cells from iWAT tissues. Differentiation or undifferentiation was conducted in primary iWAT-SVF from *Smyd2*^+/−^ and WT mice. As shown in Fig. 3G, the expressions of Smyd2, adipogenesis markers (PPARγ and C/EBPα), and the adipogenesis rate-limiting enzymes (ACC and FASN) were significantly downregulated in *Smyd2*^+/−^-iWAT-SVF upon differentiation compared with WT-iWAT-SVF. Collectively, these above data suggested that Smyd2 deficiency inhibited adipocyte differentiation in vitro.

### LLY-507, a potent and selective inhibitor of Smyd2, restrained adipocyte differentiation in vitro

**Fig. 4:**
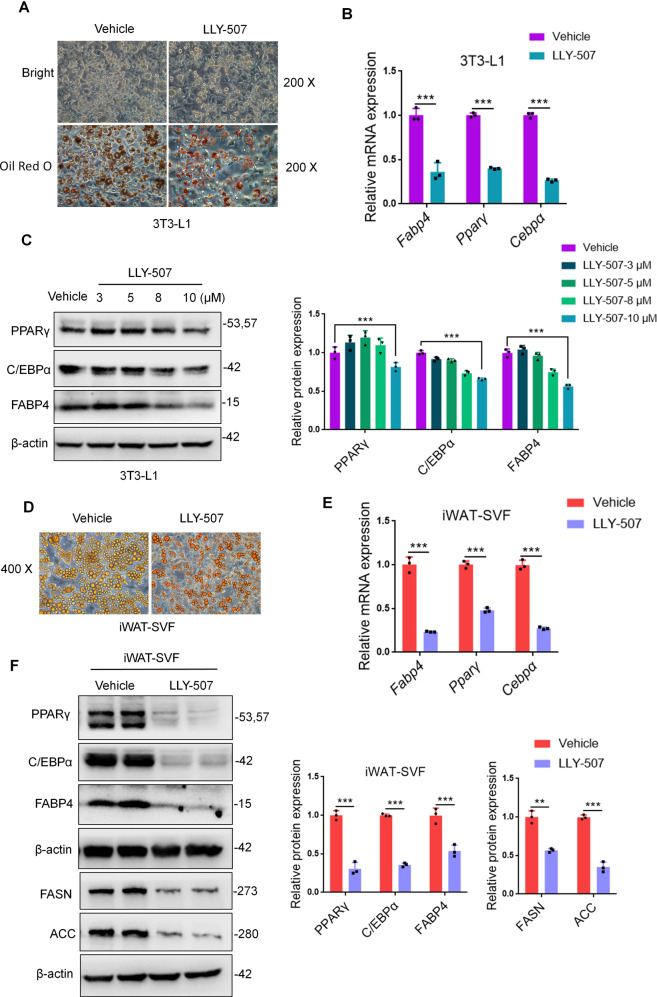
LLY-507, a potent and selective inhibitor of Smyd2, restrains adipocyte differentiation in vitro. 3T3-L1 preadipocytes or iWAT-SVF were treated with LLY-507 and meanwhile differentiated into mature adipocytes. **A** The Oil Red O staining of 3T3-L1 adipocytes. Magnification ×200. **B** The mRNA levels of adipogenesis markers (*Pparγ*, *Cepbα*, and *Fabp4*) in 3T3-L1 adipocytes. **C** The protein expressions of adipogenesis markers (PPARγ, C/EBPα, and FABP4) in 3T3-L1 adipocytes treated with LLY-507 in a dose-dependent manner. **D** The Oil Red O staining of iWAT-SVF differentiated adipocytes. Magnification ×400. **E** The mRNA levels of adipogenesis markers (*Pparγ*, *Cepbα* and *Fabp4*) in iWAT-SVF differentiated adipocytes. **F** The protein expressions of adipogenesis markers (PPARγ, C/EBPα and FABP4) and the rate-limiting enzyme in adipogenesis (ACC and FASN) in iWAT-SVF differentiated adipocytes. Data are presented as mean ± SD. **p < 0.01, ***p < 0.001.

LLY-507 has been reported to be a potent and selective inhibitor of Smyd2 [21] but whether LLY-507 restrained adipocyte differentiation in vitro has not been proven clearly. Here, 3T3-L1 preadipocytes or iWAT-SVF were treated with LLY-507 and meanwhile performed differentiation. It is noteworthy that the adipocytes differentiation efficiency was dramatically decreased in LLY-507-treated adipocytes as evidenced by fewer lipid droplets formation under bright field and Oil Red O staining (Fig. 4A). In addition, the mRNA and protein levels of adipogenesis markers (PPARγ, C/EBPα, and FABP4) were significantly decreased in LLY-507-treated 3T3-L1 preadipocytes (Fig. 4B, C). To further assess, we obtained similar patterns in LLY-507-treated iWAT-SVF detected by the Oil Red O staining, RT-qPCR, and western blot (Fig. 4D–F), suggesting that the potent and selective inhibitor of Smyd2, LLY-507, was able to restrain adipocyte differentiation in vitro.

### Smyd2 regulates adipocyte differentiation via phosphorylating STAT3

**Fig. 5:**
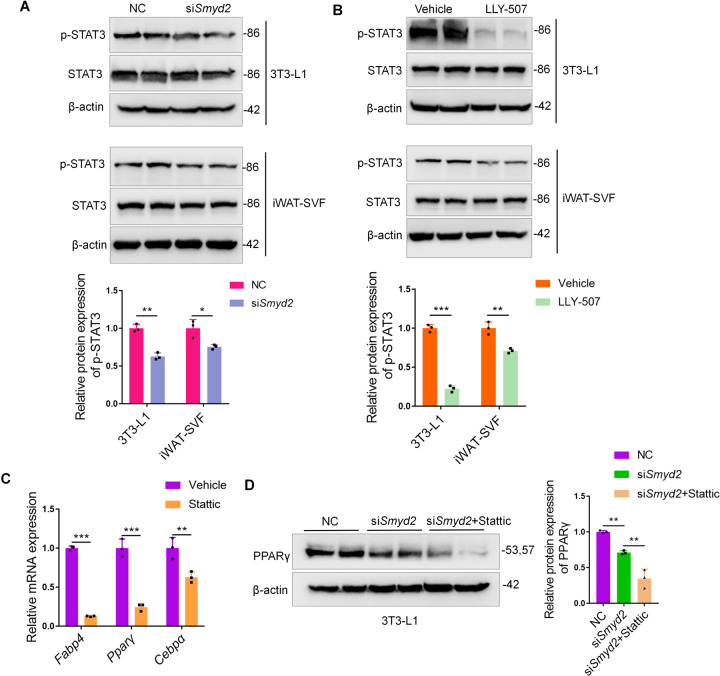
Smyd2 regulates adipocyte differentiation via phosphorylating STAT3. **A, B** The phosphorylation levels of STAT3 (Tyr705), as well as the unphosphorylated form, were detected by western blot in either Smyd2 siRNA- or LLY-507-treated 3T3-L1 and iWAT-SVF adipocytes. **C** The RT-qPCR analysis of the adipogenesis marker genes (*Pparγ*, *Cepbα*, and *Fabp4*) in Stattic (5 μM)-treated 3T3-L1 adipocytes. **D** The PPARγ protein expression was detected by western blot upon *Smyd2* siRNA transfection or *Smyd2* siRNA in combination with Stattic (5 μM) treatment. Data are presented as mean ± SD. *p < 0.05, **p < 0.01, ***p < 0.001.

Given that Smyd2 could methylate and activate the transcription factor STAT3 in autosomal dominant polycystic kidney disease [17] and adipogenesis is intimately linked to STAT3 [22], we were curious whether Smyd2 regulates adipocyte differentiation via activation of STAT3. Here we detected the phosphorylation level of STAT3 during the 3T3-L1 cell or iWAT-SVF differentiation with either *Smyd2* siRNA or LLY-507 treatment. As shown in Fig. 5A, B, the phosphorylation of STAT3 (Tyr705) significantly decreased but the expressions of total-STAT3 were not disturbed in either *Smyd2* siRNA- or LLY-507-treated 3T3-L1 cells or iWAT-SVF. Moreover, we used Stattic, a small molecule inhibitor of STAT3, which effectively inhibits STAT3 activation and nuclear translocation [23], to detect whether inhibition of STAT3 could ablate adipocyte differentiation. The RT-qPCR analysis showed that the adipogenesis marker genes (PPARγ, C/EBPα, and FABP4) were significantly decreased in Stattic-treated 3T3-L1 cells (Fig. 5C). Furthermore, to explore whether the Smyd2 siRNA-mediated adipogenesis inhibition is via STAT3 phosphorylation decrease, we combined the *Smyd2* siRNA and Stattic treatment to analyze the expression changes of PPARγ protein during the 3T3-L1 preadipocyte differentiation. Surprisingly, PPARγ protein expression was downregulated upon *Smyd2* siRNA transfection and further lower in the *Smyd2* siRNA + Stattic treatment group (Fig. 5D), suggesting that STAT3 is required for the significant reduction in PPARγ protein levels in Smyd2 knockout cells to determine the fate of adipocyte differentiation.

### *Smyd2*^+/−^ mice exert significant fat loss

**Fig. 6:**
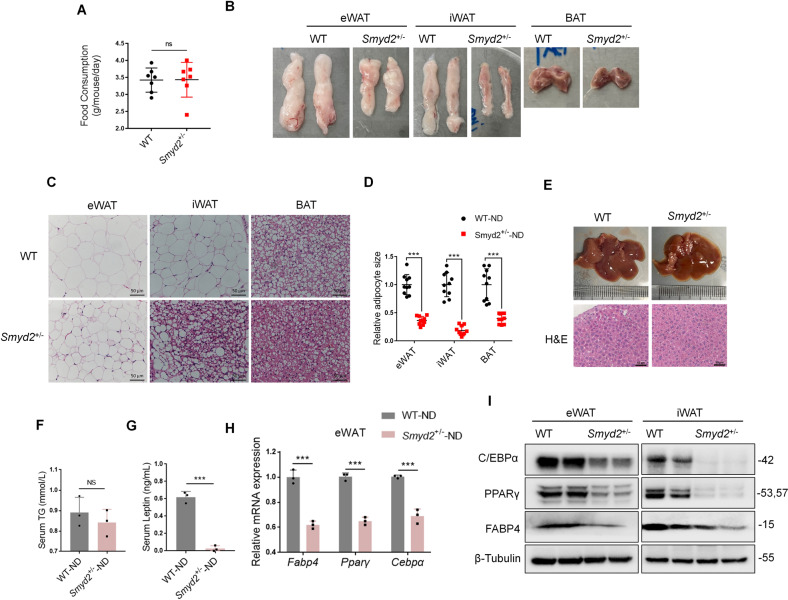
*Smyd2*^+/−^ mice exert significant fat loss. The Smyd2 heterozygous knockout (*Smyd2*^+/−^) mice and the littermate controls (WT) were obtained as we described previously [20]. **A** The food intake of *Smyd2*^+/−^ mice and their littermate controls under the normal diet. **B** The representative images of gross morphology of eWAT, iWAT and BAT from both WT and *Smyd2*^+/−^ mice. *n* = 3 mice/group. **C, D** The paraffin-embedded sections of eWAT, iWAT, and BAT from both WT and *Smyd2*^+/−^ mice were subjected to H&E staining and then the adipocyte areas were measured. Scale bar, 50 μm. **E** The representative gross morphology images and H&E staining images of liver from both WT and *Smyd2*^+/−^ mice. Scale bar, 50 μm. **F, G** The serum TG and leptin levels of WT and *Smyd2*^+/−^ mice, *n* = 3. **H** The mRNA levels of adipogenesis marker genes (*Pparγ*, *Cebpα*, and *Fabp4*) in eWAT from WT and *Smyd2*^+/−^ mice were detected by RT-qPCR. **I** The protein expressions of PPAR*γ*, C/EBP*α*, and FABP4 of eWAT and iWAT from WT and *Smyd2*^+/−^ mice were detected by western blot analysis. Data are presented as mean ± SD. ***p < 0.001.

To explore whether Smyd2 is required during adipogenesis in vivo, we first verified the Smyd2 gene was successfully deleted in adipose tissues, including eWAT, iWAT and BAT by immunoblotting assays (Fig. S2). Then we monitored the food intake of *Smyd2*^+/−^ mice and their littermate controls at 4 weeks old under the normal diet and found there was no difference in food intake between the two genotypes (Fig. 6A). To further assess, we found that *Smyd2*^+/−^ mice had smaller eWAT, iWAT and BAT depots than WT mice, especially iWAT (Fig. 6B). Furthermore, the histological analysis of eWAT, iWAT and BAT indicated that the area of adipocytes of *Smyd2*^+/−^ mice was much smaller than those of WT mice (Fig. 6C, D). To investigate whether the fat loss of *Smyd2*^+/−^ mice results in fatty liver, we harvested the liver tissue. Compared with the littermate controls, *Smyd2*^+/−^ mice did not develop into the severe fatty liver, which was also examined by the histological analysis (Fig. 6E).

Taking it a step further, we also detected the changes in the serum triglyceride (TG) and leptin levels in WT and *Smyd2*^+/−^ mice and found there was no significant difference in serum TG (Fig. 6F). However, the serum leptin level of *Smyd2*^+/−^ mice was profoundly decreased compared to that of WT mice (Fig. 6G). To further explore the potential mechanism of the significant fat loss of *Smyd2*^+/−^ mice, the mRNA levels of adipogenesis marker genes (*Pparγ*, *Cebpα*, *Fabp4*) in eWAT from WT and *Smyd2*^+/−^ mice were detected, and we found these genes were significantly reduced in *Smyd2*^+/−^ mice (Fig. 6H). In addition, the decreased expressions of PPAR*γ*, C/EBP*α*, and FABP4 of eWAT and iWAT from *Smyd2*^+/−^ mice were also verified by western blot analysis (Fig. 6I). Altogether, these above results showed that *Smyd2*^+/−^ mice exerted significant fat loss, demonstrating that Smyd2 may have regulatory effects on adipogenesis in vivo.

### *Smyd2*^+/−^ mice are not susceptible to HFD-induced obesity

**Fig. 7:**
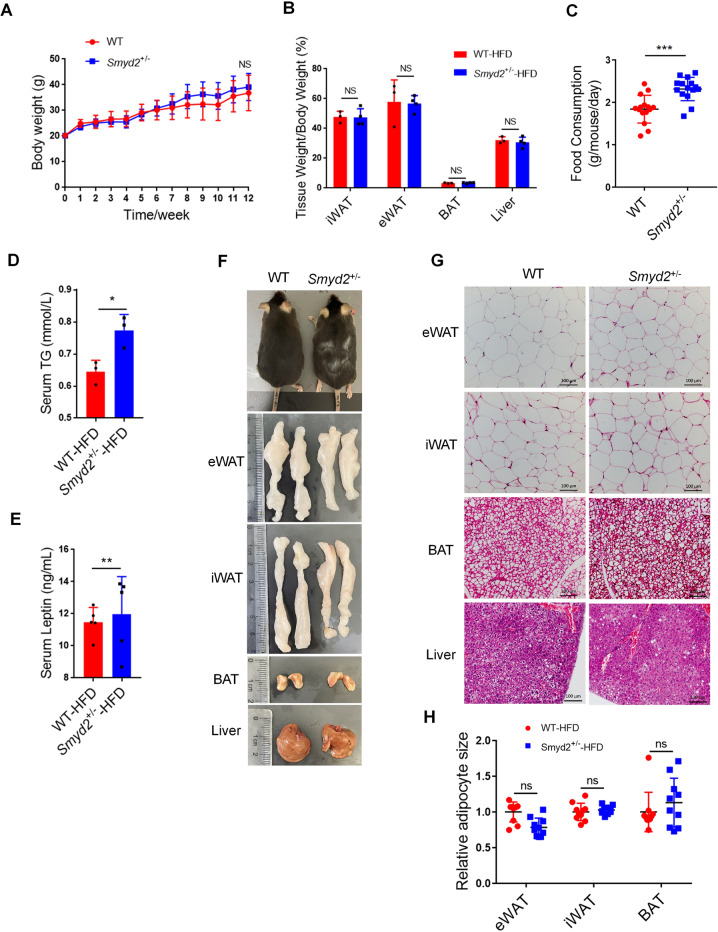
*Smyd2*^+/−^ mice are not susceptible to HFD-induced obesity. **A** Body weights of HFD-fed WT and *Smyd2*^+/−^ mice were measured each week for 12 weeks (*n* = 7). **B** The ratio of fat pad weight and body weight of HFD-fed WT and *Smyd2*^+/−^ mice. **C** The food consumption of WT and *Smyd2*^+/−^ mice upon HFD for 12 weeks. **D, E** The serum TG and leptin levels were detected after 12 weeks of HFD feeding. **F** Representative images of mice and the fat pads and liver from WT and *Smyd2*^+/−^ mice after 12 weeks of HFD feeding. **G, H** Histological analysis of eWAT, iWAT, BAT, and liver from WT and *Smyd2*^+/−^ mice after 12 weeks HFD feeding and then the adipocyte areas were measured. Scale bar, 100 μm. Data are presented as mean ± SD. *p < 0.05, **p < 0.01, ***p < 0.001.

Next, WT and *Smyd2*^+/−^ mice were subjected to a 60% HFD for 12 weeks to investigate the effects of Smyd2 on HFD-induced obesity. The body weights of mice were monitored each week and we found there was no significant change between WT and *Smyd2*^+/−^ mice (Fig. 7A). Meanwhile, the fat pads and liver weights of the HFD-fed *Smyd2*^+/−^ mice also had no difference compared with those of WT mice (Fig. 7B). Next the fasting blood glucose levels, glucose tolerance, and insulin tolerance were examined and we found no difference between HFD-fed WT and *Smyd2*^+/−^ mice (Fig. S3A–C). However, interestingly, the food intake per day, the serum TGs and leptin levels of HFD-fed *Smyd2*^+/−^ mice were much higher than those of the WT mice (Fig. 7C–E).

To further assess, the morphological analysis of HFD-fed WT and *Smyd2*^+/−^ mice and the fat pads and liver were shown in Fig. 7F. Consistent with the results mentioned above, the body size and weight of the fat pads and liver from WT and *Smyd2*^+/−^ mice had no obvious change. These results were also confirmed by H&E staining which showed the similar adipocyte size of eWAT, iWAT, and BAT from *Smyd2*^+/−^ mice compared with those of WT mice (Fig. 7G, H). Then we examined the mRNA and protein expressions of adipogenesis markers (PPAR*γ*, C/EBP*α*, and FABP4). As shown in Fig. S3D, the mRNA level of the *Cebpα* gene was significantly upregulated, while the mRNA levels of *Fabp4* genes were decreased in eWAT from HFD-fed *Smyd2*^+/−^ mice compared to WT mice. But in iWAT and BAT, there were slight changes in the expression of adipogenesis marker genes (*Pparγ*, *Cebpα, and Fabp4*) between WT and *Smyd2*^+/−^ mice. Strangely, the protein levels of adipogenesis markers (PPAR*γ*, C/EBP*α*, and FABP4) showed a slight upregulation trend in eWAT and iWAT from *Smyd2*^+/−^ mice (Fig. S3E), suggesting that the expressions of adipogenesis markers in HFD-induced *Smyd2*^+/−^ mice showed inconsistent trends. These seemingly contradictory trends may be due to the heterozygous mice (*Smyd2*^+/−^) we used, which fed by HFD exhibit a complex overall metabolic state.

### HFD-fed *Smyd2*^+/−^ mice show leptin resistance

Take a further step, we continued to explore why the food intake of HFD-fed *Smyd2*^+/−^ mice was significantly higher than that of WT mice (Fig. 7C) while there was no difference in food intake between the two genotypes under the normal diets (Fig. 6A). Studies have shown that food intake was closely related to the level of leptin, which is a protein-like hormone secreted by adipose tissues [24]. Our previous results showed that ND-fed *Smyd2*^+/−^ mice showed much less leptin level than WT mice (Fig. 6F), while after 12 weeks of HFD feeding, the serum leptin level of *Smyd2*^+/−^ mice was significantly upregulated than that of WT mice (Fig. 7E), suggesting that *Smyd2*^+/−^ mice fed by HFD may be under the leptin resistance. Therefore, we investigated the canonical JAK2-STAT3 signaling pathway, which is activated by leptin in the hypothalamus [25]. As shown in Fig. S4, the phosphorylation level of JAK2-STAT3 signaling was increased in the hypothalamus tissue of HFD-fed WT mice but decreased in HFD-fed *Smyd2*^+/−^ mice. Interestingly, the protein expression of SOCS3 was significantly upregulated in the hypothalamus of HFD-fed *Smyd2*^+/−^ mice. These data demonstrated that *Smyd2*^+/−^ mice fed by HFD could be under leptin resistance, resulting in a significantly higher food intake of *Smyd2*^+/−^ mice than that of WT mice.

## Discussion

Here, our observations show that Smyd2 is a critical regulator with the ability to regulate adipocyte differentiation and adipogenesis by phosphorylating STAT3 in vitro. Knockdown or inhibition of Smyd2 in preadipocytes restrained their ability to differentiate as detected by less Oil Red O staining and deceased adipocyte marker gene expressions. Besides, *Smyd2*^+/−^ mice fed with normal diets exert significant fat loss but are not susceptible to HFD-induced obesity due to the heterozygous phenotype. Further studies are necessary to take advantage of adipose-specific *Smyd2* knockout mice to elucidate the regulatory functions of Smyd2 in obesity in vivo.

Smyd2 is reported as a histone methyltransferase that regulates diverse cellular processes by methylating either histones or non-histones [11]. Recurrent data have demonstrated that Smyd2 plays a key role in several types of cancer [12], immune-inflammatory diseases [20], and abdominal aortic aneurysm [16]. However, the role of Smyd2 in metabolic diseases, such as obesity, has not been reported. Thus, our present study focuses on the newly discovered role of Smyd2 in adipocyte differentiation and function. We found that Smyd2 is abundant in white adipose tissue and closely correlated with adipocyte differentiation. Knockdown with *Smyd2* siRNA or inhibition of Smyd2 with a potent and selective inhibitor LLY-507 restrained intracellular lipid accumulation as observed by Oil Red O staining. As known, adipogenesis is always coordinated by several adipogenic transcription factors. In line with the effects on lipid accumulation, we also showed that knockdown or inhibition of Smyd2 could downregulate the expressions of adipogenic differentiation markers, such as PPARγ, C/EBPα and FABP4. These above data strongly support our hypothesis that Smyd2 regulates adipocyte differentiation and may play a role in metabolic diseases.

In addition to these adipogenic transcription factors mentioned above, various adipogenesis-regulatory signaling pathways have been identified. Multiple lines of evidence suggest that STAT3 plays a key role in adipogenesis via regulating C/EBPβ and PPARγ genes [22, 26]. STAT3 could be activated within 2 h upon differentiation induction, and the phosphorylated STAT3 translocates to the nucleus to bind the distal region of the C/EBPβ promoter and further regulates the transcription of C/EBPβ at the early stage of adipogenesis [22]. In addition, STAT3 in adipocytes is constitutively active in visceral obese subjects [27] and is also a crucial effector of lipolysis-driven oxidative metabolism [28]. Consistent with these studies, we here demonstrated that Smyd2-triggered adipocyte differentiation requires the phosphorylation of STAT3, then further mediates the expression of PPARγ.

Due to the heterozygous (*Smyd2*^+/−^) mice we used in the present study, we got confusing results in HFD-fed WT and *Smyd2*^+/−^ mice. These seemingly contradictory trends may be due to the knockdown efficiency of the *Smyd2* gene in *Smyd2*^+/−^ mice could not reach the ablation level in *Smyd2* siRNA-transfected cells in vitro. *Smyd2*^+/−^ mice may regulate only some of the adipogenesis marker genes in vivo, but not enough to alleviate HFD-induced obesity. On the other hand, the body weight and fat pads weights of WT and *Smyd2*^+/−^ mice showed no difference while the food intake of *Smyd2*^+/−^ mice was significantly higher than that of WT mice, implying that excessive food intake induced obesity may partially offset the weight loss caused by *Smyd2* gene knockdown.

Take it a step further, studies have shown that food intake was closely related to leptin levels [24] and the canonical JAK2-STAT3 signaling pathway could be activated by leptin in the hypothalamus and further upregulated SOCS3, which is reported as a negative feedback regulator of leptin signal [29]. Moreover, the increased SOCS3 in the hypothalamus in turn suppresses JAK2/STAT3 signaling pathway, which forms leptin resistance, leading to hyperleptinemia and obesity [30]. In the obese state, elevated circulating leptin levels may further increase the activity of JAK2/STAT3 signaling in central neurons, which in turn increases SOCS3 expression levels and further impairs the sensitivity of leptin receptors, thereby resulting in the leptin function failure [31]. Here, we found the food intake and serum leptin level of HFD-fed *Smyd2*^+/−^ mice were significantly higher than that of WT mice, suggesting that HFD-fed *Smyd2*^+/−^ mice may be under a leptin resistance state, which was also confirmed by the downregulated phosphorylation level of JAK2-STAT3 signaling and upregulated level of SOCS3 in hypothalamus tissues of HFD-fed *Smyd2*^+/−^ mice. Combining these above explorations, we believe that further studies using fat-specific knockout mice are necessary to validate the regulation of Smyd2 in HFD-fed mice, which is one of the limitations of this present study.

## Materials and methods

### Reagents

Reagent sources were as follows: Insulin, 3-isobutyl-1-methylxanthine (IBMX), rosiglitazone, and dexamethasone were from Sigma-Aldrich (St. Louis, MO). LLY-507 was purchased from Medchemexpress (Monmouth Junction, NJ). Stattic (S7024) was obtained from Selleck (Houston, USA). Antibodies were obtained from the following commercial sources: Smyd2 (Proteintech, 21290-1-AP), p-JAK2 (Cell Signaling Technology, 3776S), p-STAT3 (Tyr705) (Cell Signaling Technology, 9134T), JAK2 (Cell Signaling Technology, 3230T), STAT3 (Cell Signaling Technology, 9139T), SOCS3 (Bioworld, BS60384), β-Tubulin (Proteintech, 66240-1-Ig), β-actin (Proteintech, 66009-1-Ig), Hsp90 (Proteintech, 13171-1-AP), C/EBPα (Cell Signaling Technology, 8178T), PPARγ (Cell Signaling Technology, 2435T), FABP4 (Cell Signaling Technology, 2120T), Fatty Acid Synthase (Cell Signaling Technology, 3180T), Acetyl-CoA Carboxylase (Cell Signaling Technology, 3676T).

### Animal studies and ethical statement

All the animal procedures were performed following the guiding principles of laboratory animal care set by the Institutional Animal Care and Use Committee (IACUC) of Fudan University. The mice were housed in the Animal Experiment Center of Fudan University and had access to food and water ad libitum. All mice were age and sex-matched and then randomized into different groups. Only male mice were used in this study to avoid potential interference with female sexual hormones. The mice sample size was detailed in the corresponding figure legends.

Smyd2 heterozygous knockout (*Smyd2*^+/−^) mice and their littermate controls (WT) were constructed as described previously [20]. To establish the high-fat diet (HFD)-induced obese mice model, 6–8 weeks of WT and *Smyd2*^+/−^ mice were randomly assigned to treatment groups and fed with HFD (60% kcal from fat, TROPHIC Animal Feed High-Tech Co. LTD, China) for 12 weeks. The mice were finally euthanized by intraperitoneal (*i.p*.) administration of overdose pentobarbital sodium (150 mg/kg body weight), then the adipose and liver tissues were harvested for further examination.

### Measurements of serum TG levels

The serum TG level was measured by the TG detection kit (Nanjing Jiancheng Bioengineering Institute, China) according to the manufacturer’s instructions. Briefly, the serum of mice was collected and incubated with the working stock solution for 10 min at 37 °C. Then the OD values were detected at the wave of 510 nm.

### Measurements of serum leptin levels

The serum leptin level was detected by using the enzyme-linked immunosorbent assay kit for mouse leptin according to the manufacturer’s instructions (Cloud-Clone Corp. Wuhan, China). Briefly, the standard curve was established by the standard sample in a dose-dependent manner. Next, the serum samples were incubated with Detection Reagent A for 1 h and Detection Reagent B for 30 min at 37 °C, respectively. After repeating the aspiration/wash process for a total of 5 times, the samples were incubated with Substrate Solution for 10–20 minutes at 37 °C then add Stop Solution. Finally, run the microplate reader and conduct measurement at 450 nm immediately.

### Glucose tolerance test (GTT) & insulin tolerance test (ITT)

For glucose tolerance test (GTT), mice fed with HFD for 10 weeks were fasted overnight, followed by intraperitoneal glucose (1.50 g/kg) injection. For the insulin tolerance test (ITT), human insulin (Sigma-Aldrich) was injected (0.75 units/kg) into mice fed with HFD for 11 weeks. The blood glucose levels were monitored at 0, 15, 30, 60, and 120 min after injection using the one-touch glucometer.

### Histology staining analysis

The adipose or liver tissues were fixed with 4% paraformaldehyde and then embedded in paraffin. Hematoxylin/eosin (H&E) staining was performed to observe the morphological changes in each group. Representative images were captured under the ZEISS light microscope.

### Cells Culture and treatments

For isolation and differentiation of primary mouse stromal vascular fraction (SVF), the iWAT of mice were collected from *Smyd2*^+/−^ mice and their littermate controls (WT) and minced, followed by digesting in 0.2% collagenase containing 2.5% BSA for 40 min, then filtered and centrifuged to obtain stromal vascular fraction (SVF) cells in the pellets. Primary iWAT-SVF cells were cultured in DMEM/F12 with 10% fetal bovine serum (FBS) until confluence. Primary iWAT-SVF cells were induced to differentiate with DMEM/F12 containing 10% FBS, 10 μg/ml insulin, 0.5 mM 1-methyl-3-isobutylxanthine (IBMX), 0.25 μM dexamethasone, and 1 μM rosiglitazone for 2 days. Then the medium was replaced with DMEM/F12 containing 10% FBS, 10 μg/ml insulin and 1 μM rosiglitazone for the next 2 days. Then cells were cultured with DMEM/F12 containing 10% FBS for another 2 days until full differentiation.

For the culture and differentiation of 3T3-L1 preadipocytes, 3T3-L1 preadipocytes were cultured in DMEM supplemented with 10% FBS and 1% penicillin-streptomycin until confluence. Then 3T3-L1 cells were differentiated using the differentiation DMEM containing FBS 10%, 10 μg/ml insulin, 0.5 mM IBMX, and 0.25 μM dexamethasone, and the time was designated as day 0 of differentiation. Two days later, cells were maintained in DMEM supplemented with 10% FBS and 10 μg/ml insulin. Then cells were maintained in the medium until full differentiation. The medium was changed every other day.

For treatments with the inhibitor, 3T3-L1 cells or iWAT-SVF cells were treated with LLY-507 (10 μM) or a dose-dependent manner from the beginning of differentiation until the end of differentiation.

### Small interfering RNA (siRNA) transfection

For siRNA transfection, the mouse Smyd2 siRNA (sense: 5’-CACCAGUUCUACUCCAAGUTT-3’, antisense: 5’-ACUUGGAGUAGAACUGGUGTT-3’) and control siRNA (5’-UUCUCCGAACGUGUCACGUTT-3’) (GenePharma, Shanghai, China) were transfected into 3T3-L1 preadipocytes or iWAT-SVF cells at day −2 using Lipofectamine RNAiMAX transfection reagent (Invitrogen) according to the manufacturer’s protocol. After transfection for 24 h, fresh serum medium was replaced and then the cells were induced to differentiation.

### Oil Red O staining

Differentiated adipocytes seeded in 24-well plates were fixed with 4% (v/v) paraformaldehyde (PFA) for 30 min and then washed 3 times with PBS. 0.5% Oil Red O (v/v) in isopropanol was diluted with ddH_2_O (3:2) and then filtered to incubate the fixed adipocytes for 1 h at RT. The stained adipocytes were observed using a microscope (Zeiss, Germany). The bright fields were photographed using a microscope (Nikon, Japan) before the Oil Red O staining.

### Western blot

Western blot analysis was performed as we previously described. Briefly, the differentiated cells or adipose tissues were homogenized in lysis buffer (LDS Sample Buffer or RIPA buffer, respectively). The whole protein samples were acquired and then separated using SDS-PAGE. Separated proteins were transferred to nitrocellulose (NC) membranes and then incubated with various primary antibodies at 4 °C overnight. The anti-rabbit or anti-mouse IgG HRP-conjugated secondary antibodies were used and then detected by ChemiDoc^+^ (Bio-RAD).

### qRT-PCR analysis

Total RNA was extracted from the differentiated cells or adipose tissues using TRIzol Reagent (TaKaRa Biotechnology, Dalian, China) and was reversely transcribed into cDNA by using a PrimeScript 1st Strand cDNA Synthesis Kit (TaKaRa Biotechnology, Dalian, China). Quantitative real-time reserve transcription PCR (qRT-PCR) was performed using the iCycler iQ system (Bio-Rad, Hercules, CA, USA) with the SYBR Green and primers then obtained the relative quantitation of the mRNA levels of target genes that were normalized to *18S*. The primer sequences of RT-qPCR were seen in Supplemental Table I.

### Statistical analysis

Quantitative data are expressed as Means ± SD. Differences in means were analyzed by using one-way or two-way ANOVA with Bonferroni’s *post hoc* test for multiple groups and an unpaired *t*-test for two groups. Probability values, *p* < 0.05 was considered statistically significant. GraphPad Prism 7.0 software (GraphPad Software Inc., San Diego, CA, US) was used for all quantitative analyses.

## Supplementary information

SUPPLEMENTAL MATERIAL

aj-checklist

Supplemental Material-WB

## Data Availability

All data generated or analyzed during this study are included in this published article [and its supplementary information files].
