# Supplementary material for: Histone methyltransferase Smyd2 drives adipogenesis via regulating STAT3 phosphorylation
Source: Cell Death Dis. 2022 Oct 21;13(10):890. doi: 10.1038/s41419-022-05321-7 (PMC9586978; doi:10.1038/s41419-022-05321-7)
Supplement: Supplementary file 1 — SUPPLEMENTAL MATERIAL [file 41419_2022_5321_MOESM1_ESM.docx]

**Supplemental Figures**


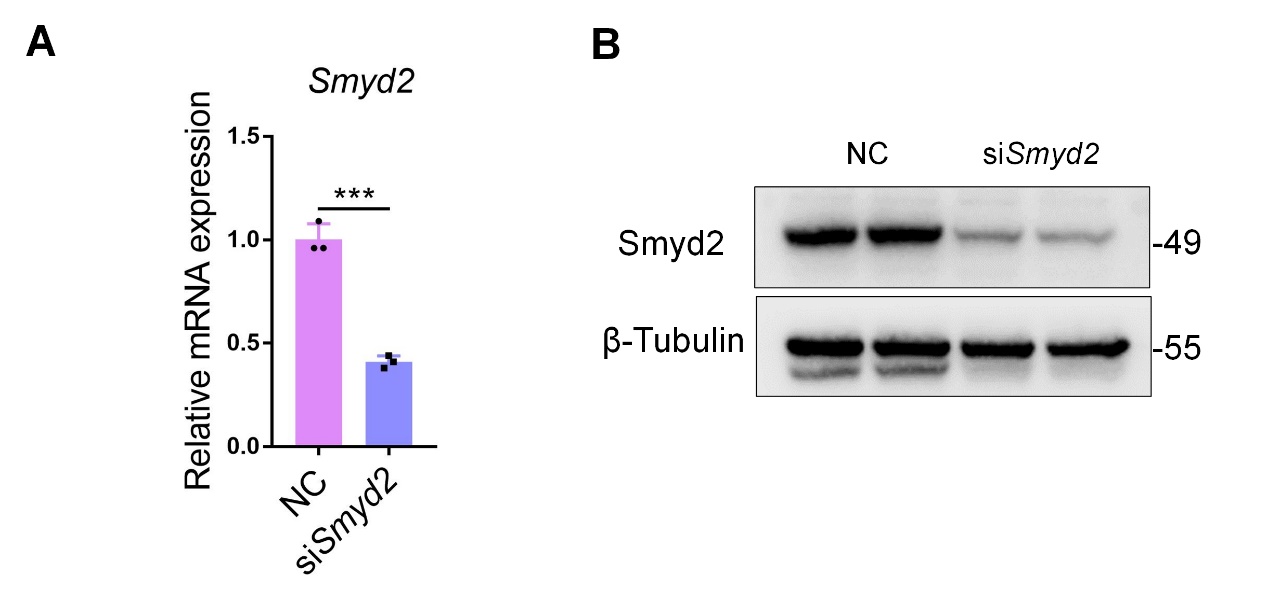


**Figure S1.** **The knockdown efficiency of *Smyd2* siRNA.** (**A-B**) The mRNA and protein expression of Smyd2 in NC- and *Smyd2* siRNA-transfected 3T3-L1 cells. NC, the negative control siRNA. Data are presented as mean ± SD. ****p* < 0.001.


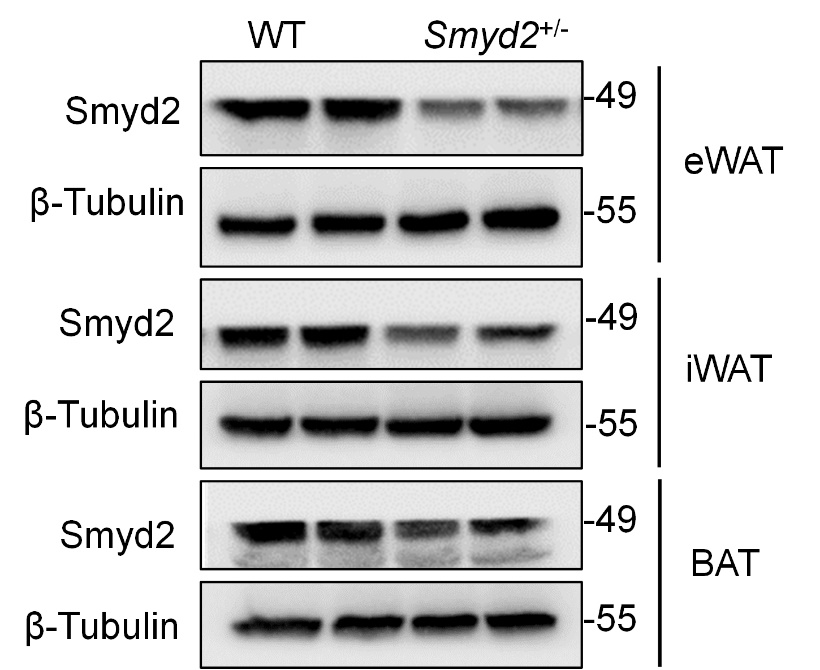


**Figure S2.** **The verification of Smyd2 knockdown in adipose tissues from *Smyd2*^+/-^ and WT mice.** The protein expressions of Smyd2 in adipose tissues (eWAT, iWAT and BAT) from *Smyd2*^+/-^ and WT mice were detected by immunoblotting assay. n = 3 mice/group.


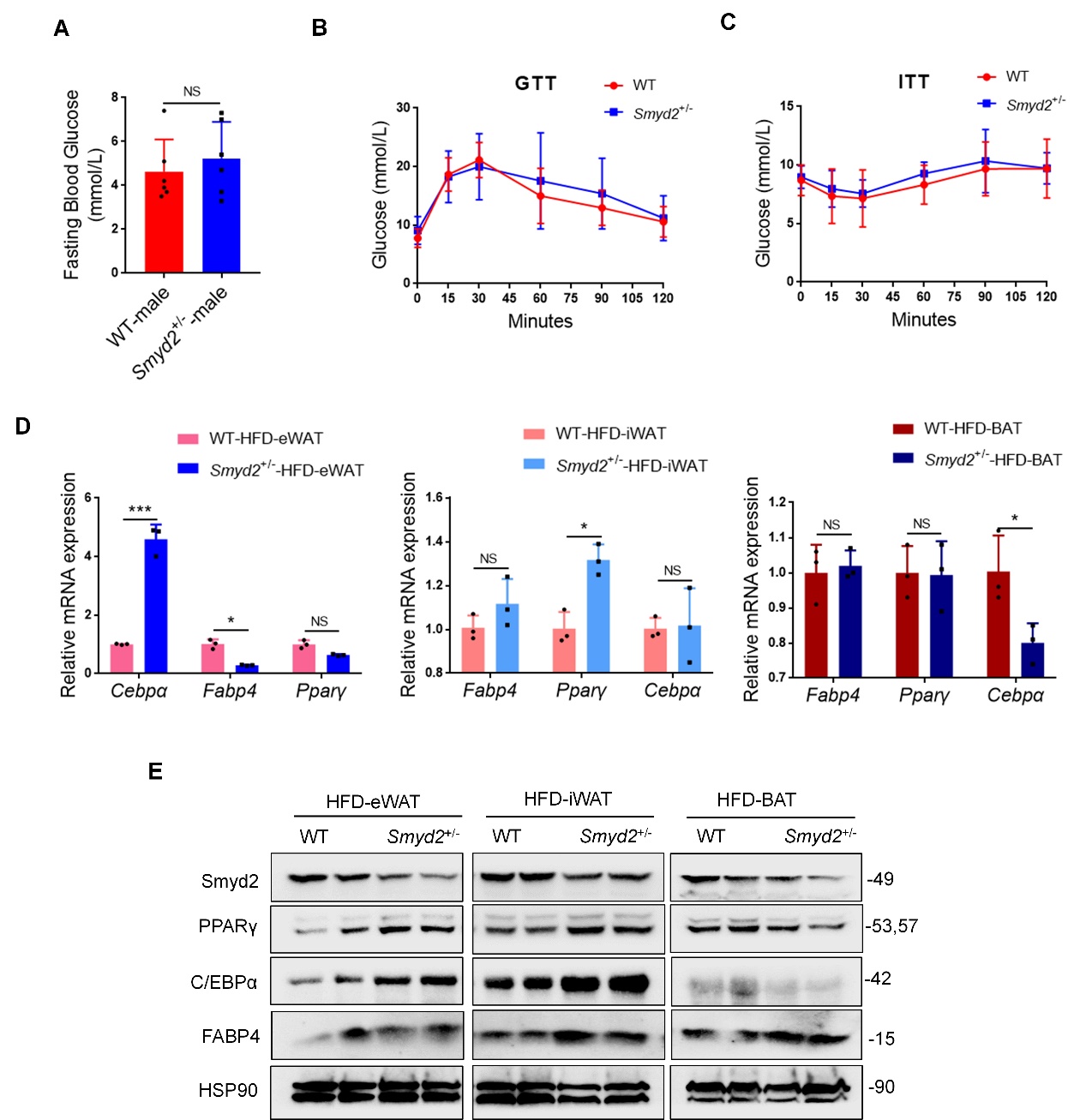


**Figure S3.** **HFD-fed *Smyd2*^+/-^ mice show no significant change in** **physiological indicators and adipogenesis markers.** (**A**) The levels of fasting blood glucose of WT and *Smyd2*^+/-^ mice after 12 weeks of HFD feeding. (**B**) Glucose tolerance test (GTT) was performed after mice were fed with HFD for 10 weeks. (**C**) Insulin tolerance test (ITT) was performed after mice were fed with HFD for 11 weeks. (**D-E**) The mRNA and protein levels of adipogenesis marker genes (*Pparγ*, *Cebpα, and* *Fabp4*) of eWAT, iWAT and BAT from WT and *Smyd2*^+/-^ mice after 12 weeks of HFD feeding. Data are presented as mean ± SD. **p* < 0.05, ****p* < 0.001.


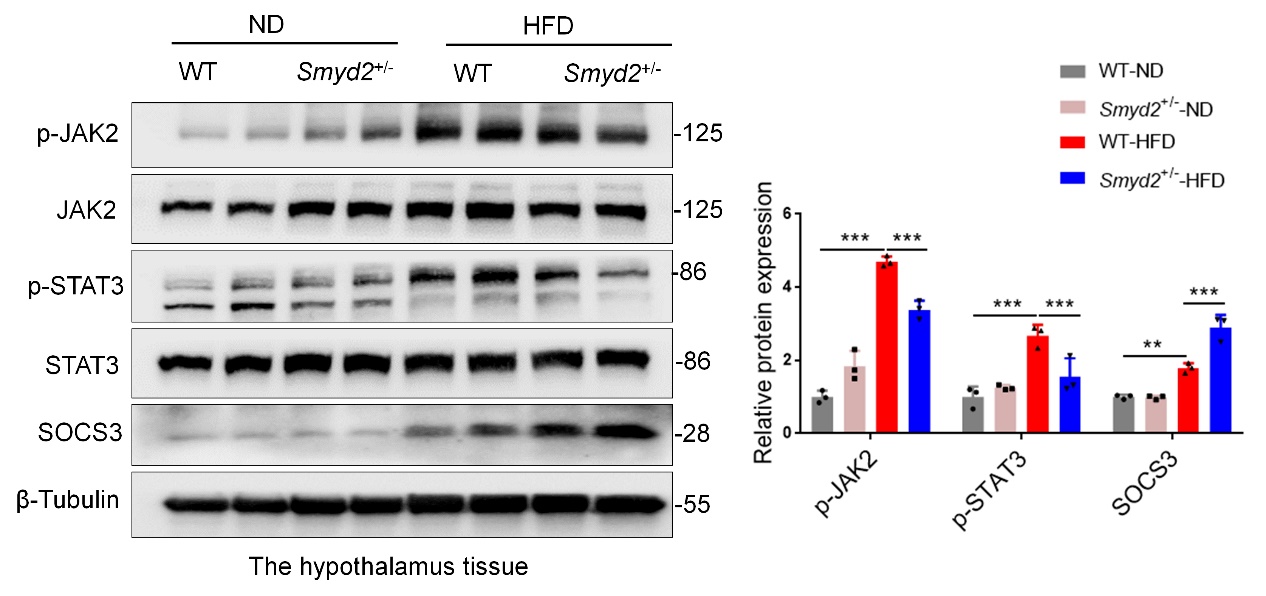


**Figure S4. HFD-fed *Smyd2*^+/-^ mice represent leptin resistance.** The protein levels of p-JAK2, JAK2, p-STAT3, STAT3 and SOCS3 in hypothalamus from WT and *Smyd2*^+/-^ mice after the absence or presence of HFD feeding. Data are presented as mean ± SD. ***p* < 0.01, ****p* < 0.001.

**SUPPLEMENTAL TABLE**

Table S1. The primer sequences used for qPCR analysis.

| Gene | Sequence |
| --- | --- |
| Mus-*Smyd2*-F | ACAGAAGACAGGAACGACCG |
| Mus-*Smyd2*-R | ATGTCTCGGATGGCTTCTGC |
| Mus-*Cebpα*-F | CAAGAACAGCAACGAGTACCG |
| Mus-*Cebpα*-R | GTCACTCGTCAACTCCAGCAC |
| Mus-*Pparγ*-F | GTGCCAGTTTCGATCCGTAGA |
| Mus-*Pparγ*-R | GGCCAGCATCGTGTAGATGA |
| Mus-*Fabp4*-F | GATGCCTTTGTGGGAACCT |
| Mus-*Fabp4*-R | CTGTCGTCTGCGGTGATTT |
| Mus-*18S*-F | CGCCGCTAGAGGTGAAATTCT |
| Mus-*18S*-R | CATTCTTGGCAAATGCTTTCG |
