# Supplementary figures and images for: Histone methyltransferase Smyd2 drives adipogenesis via regulating STAT3 phosphorylation
Source: Cell Death Dis. 2022 Oct 21;13(10):890. doi: 10.1038/s41419-022-05321-7 (PMC9586978; doi:10.1038/s41419-022-05321-7)

Figure 1

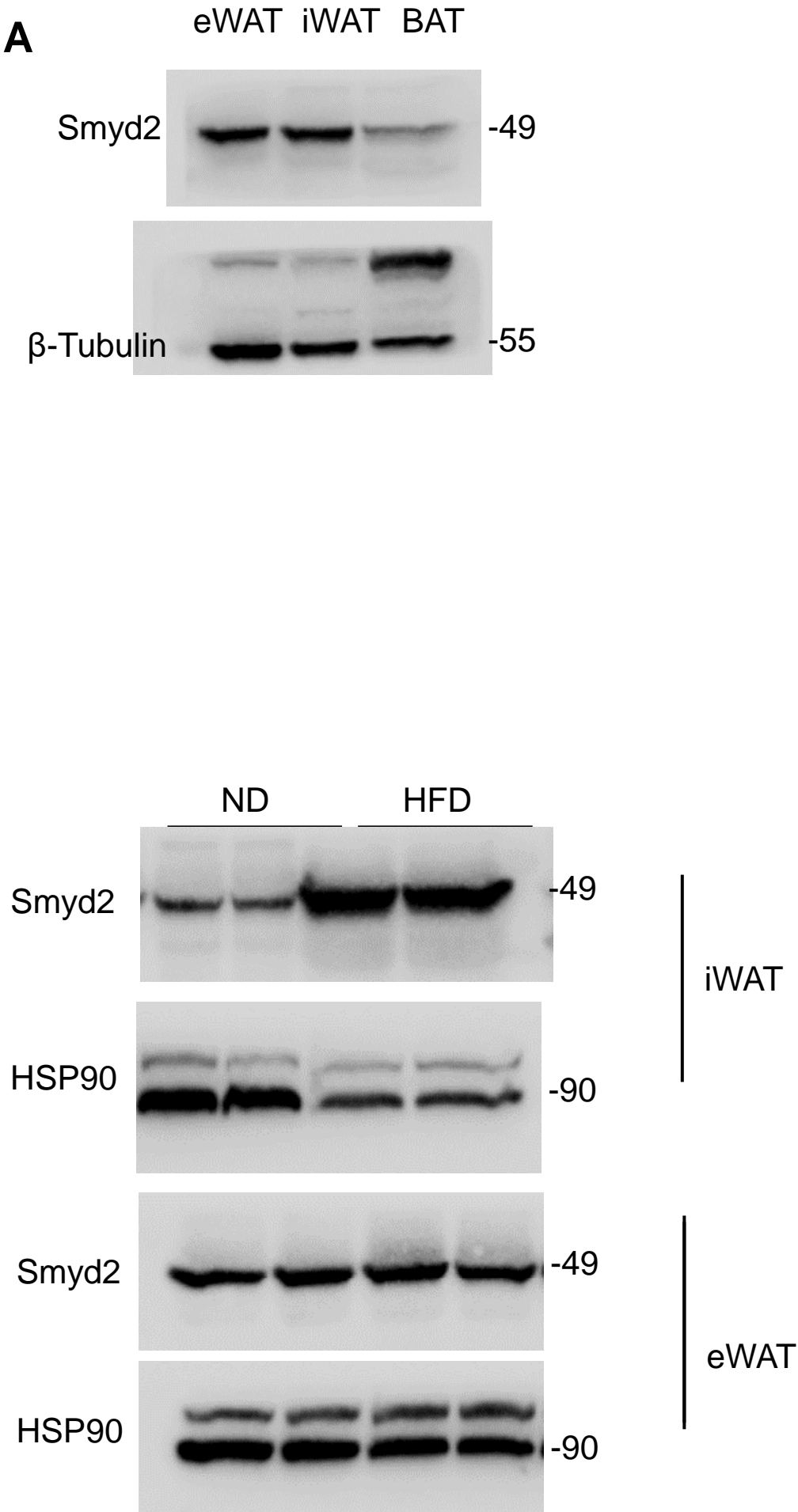

Figure 2

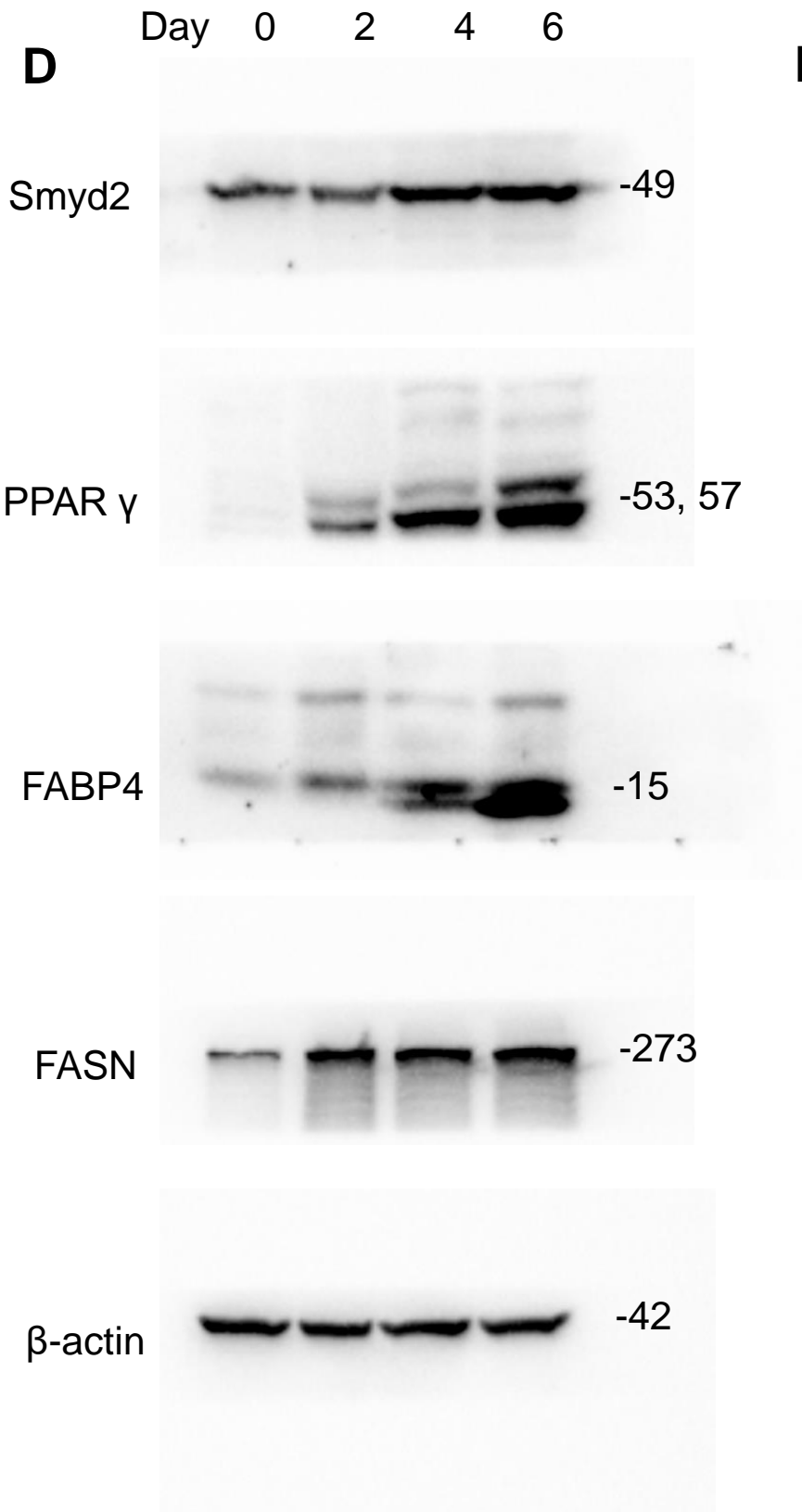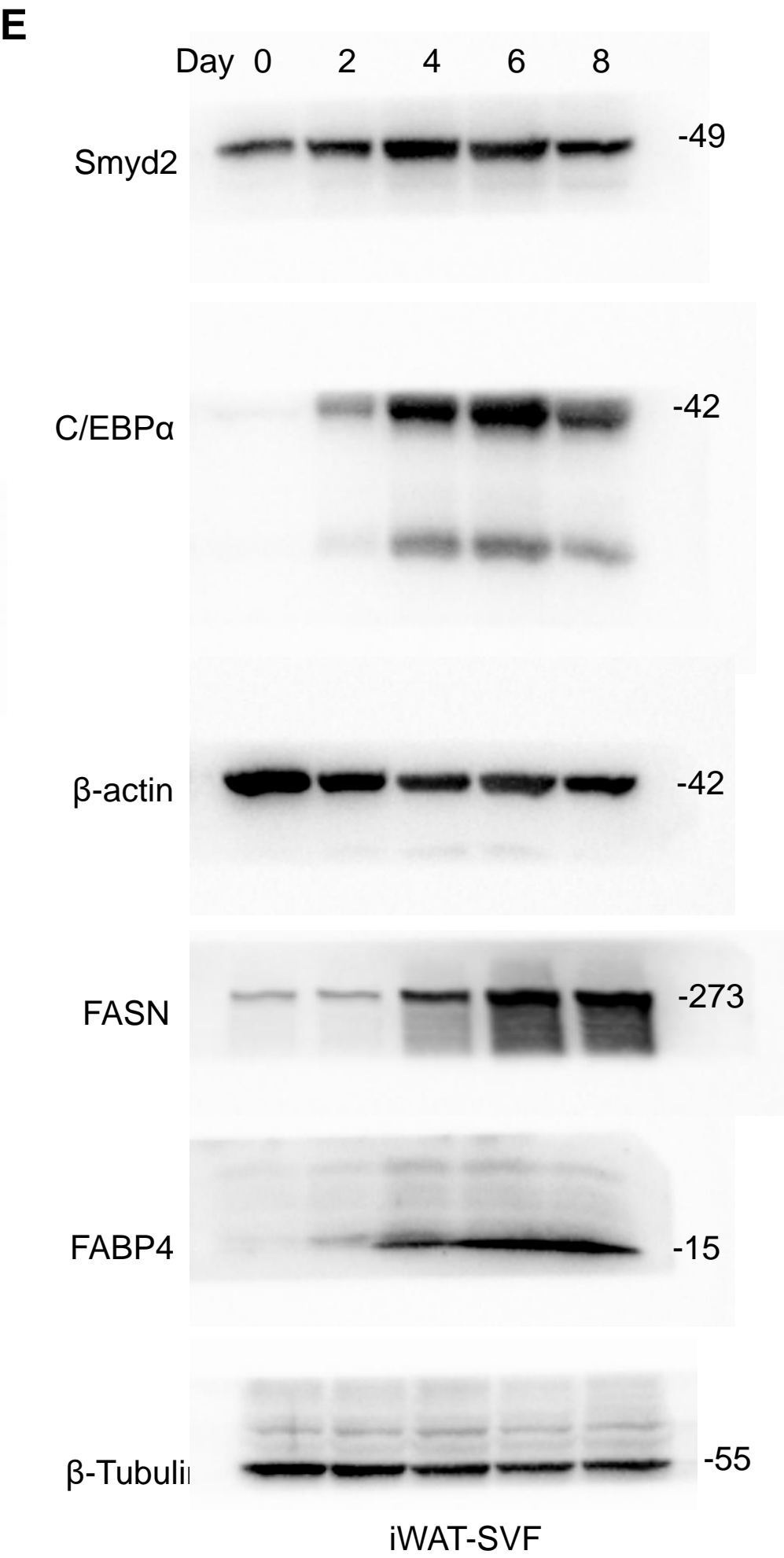

Figure 3

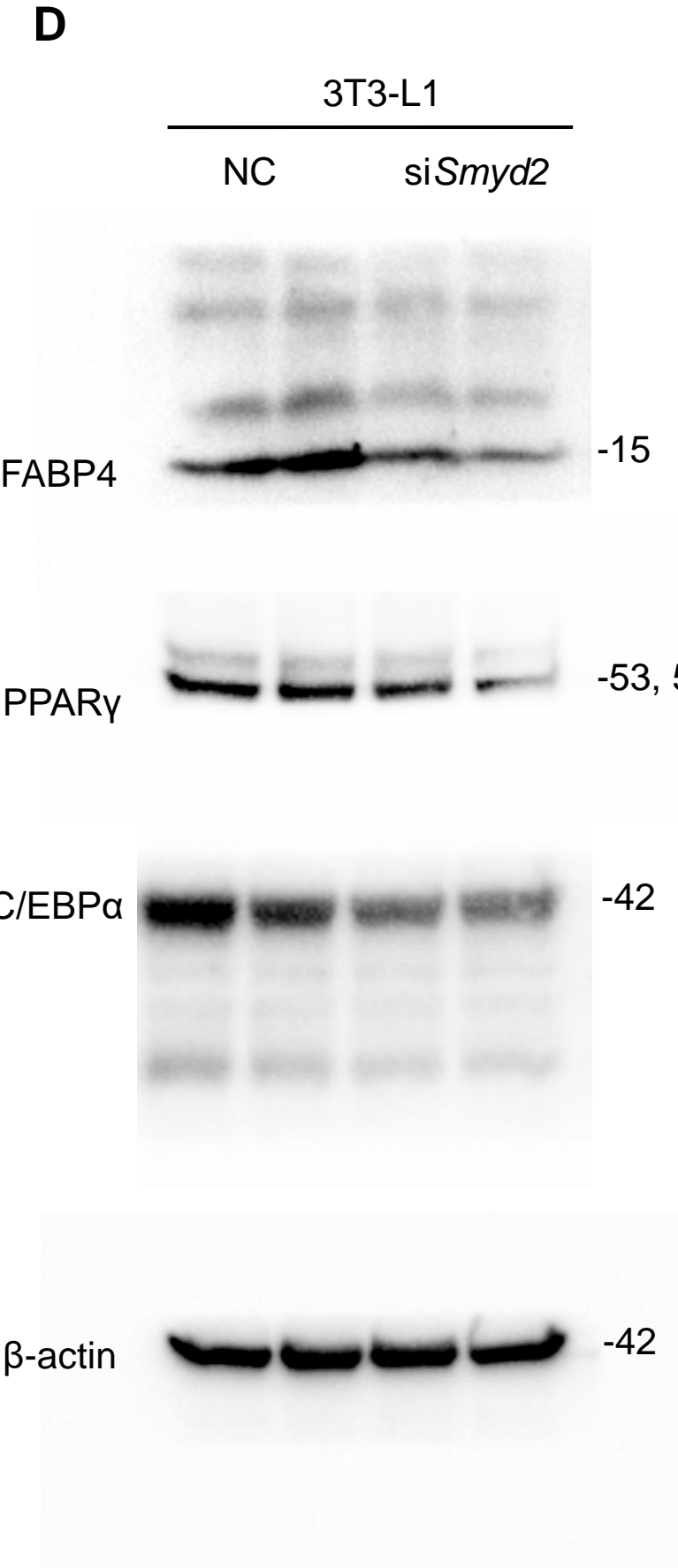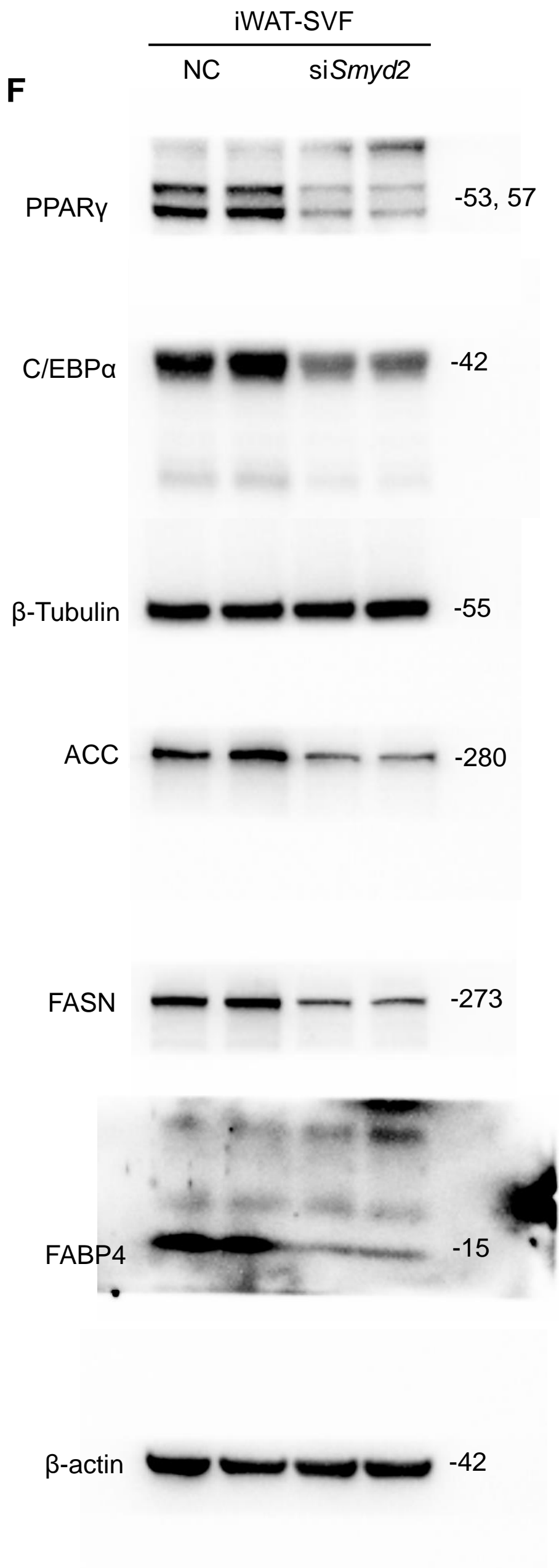

Figure 3

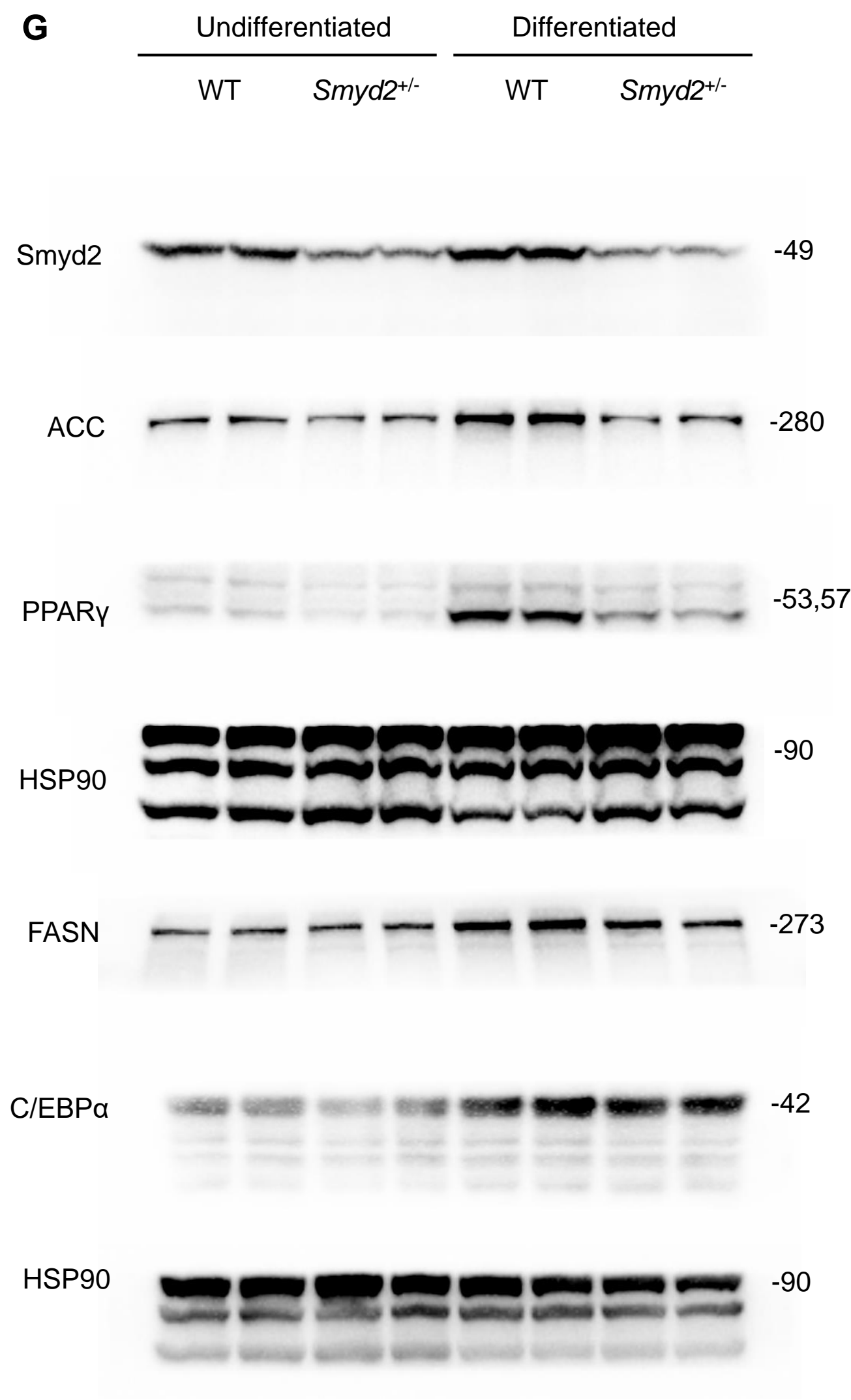

Figure 4

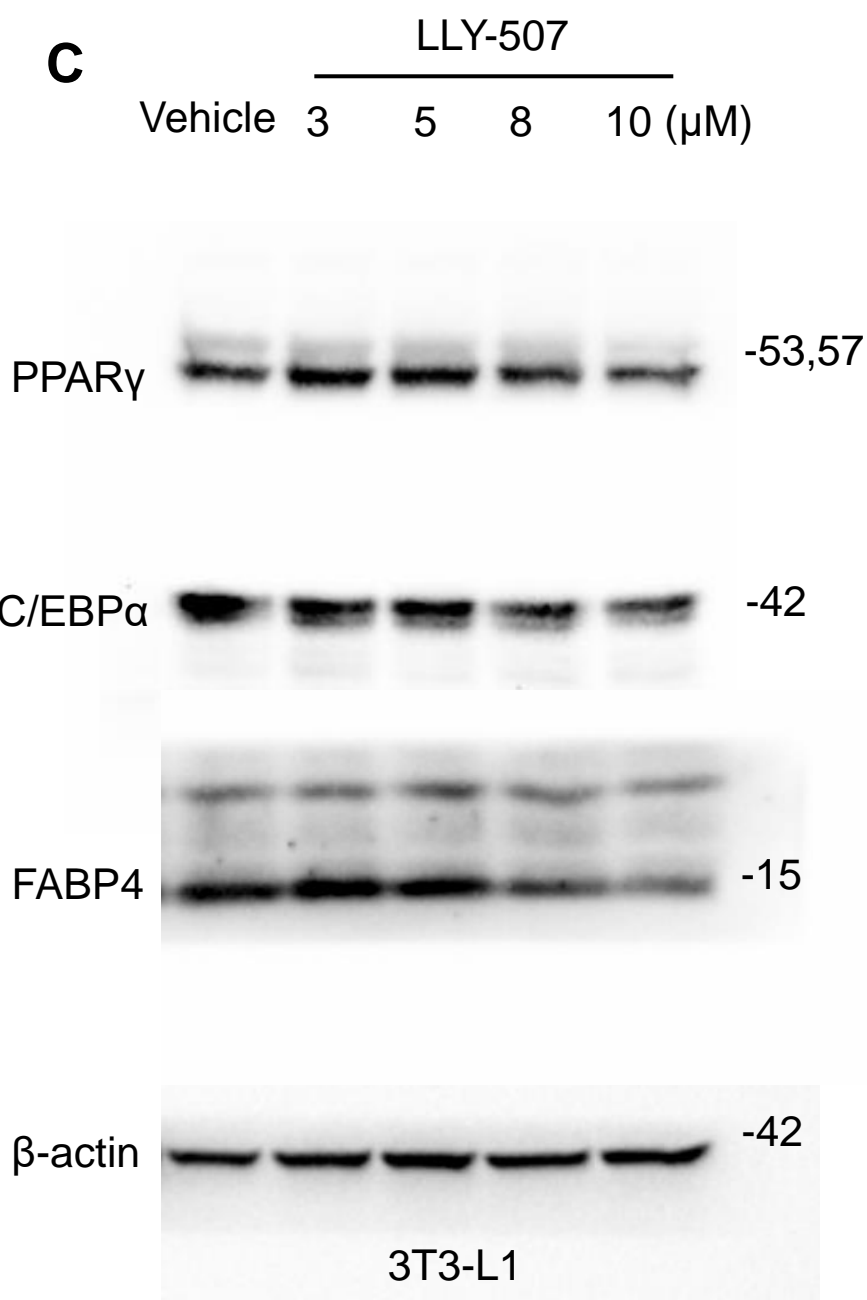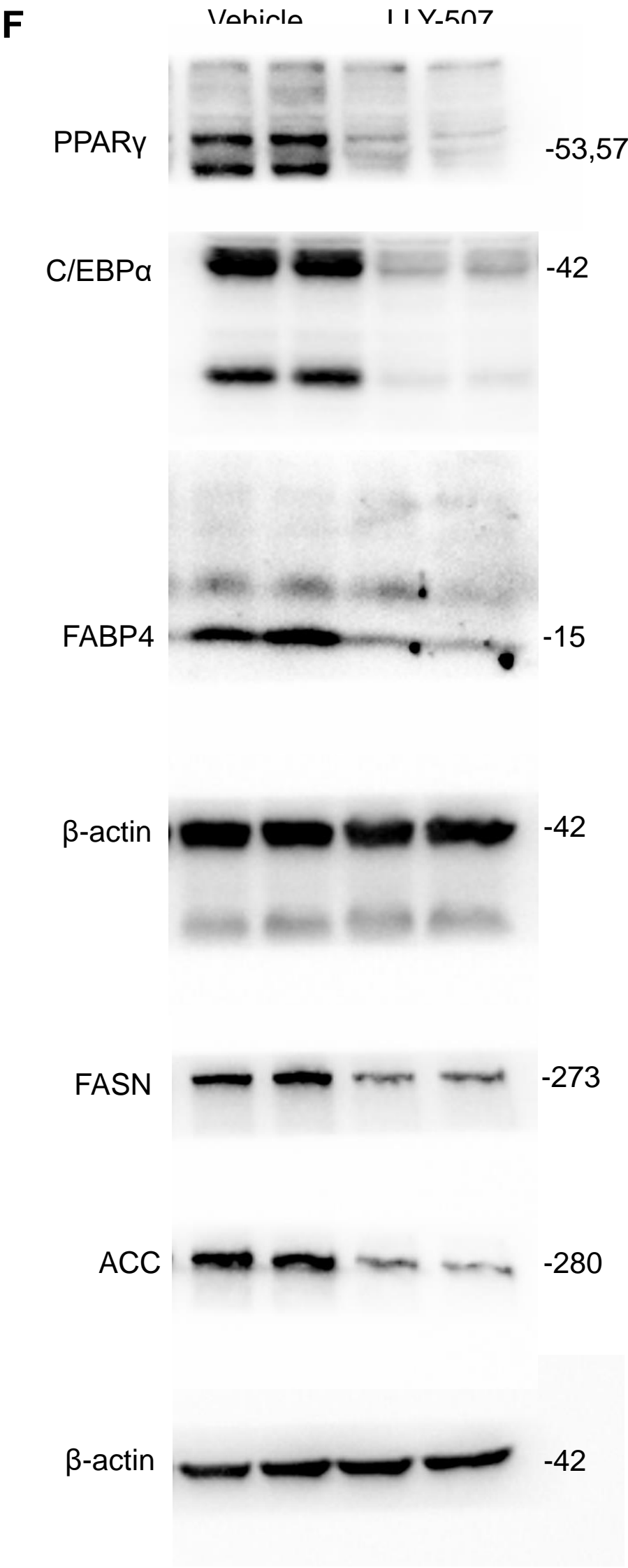

Figure 5

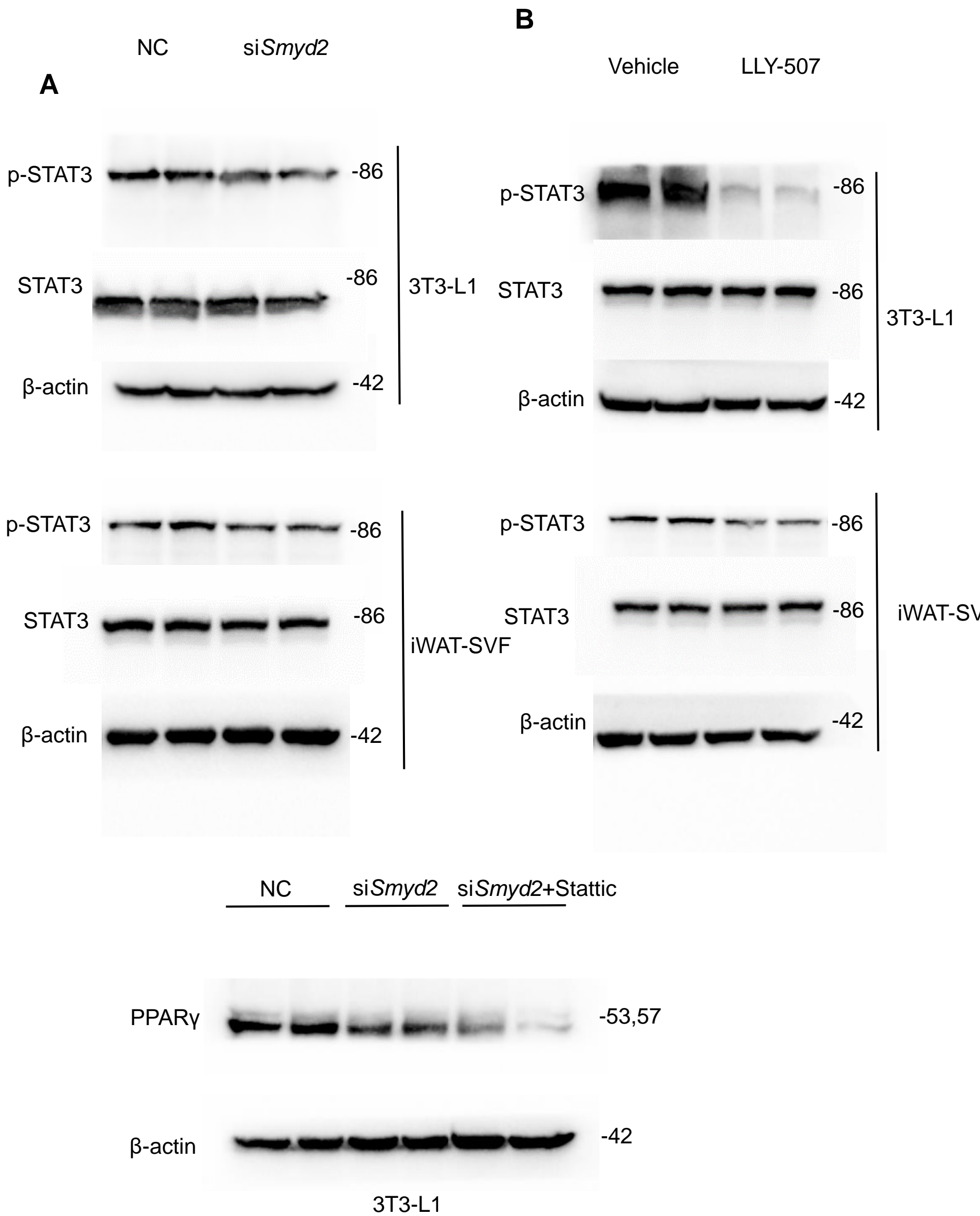

Figure 6

I

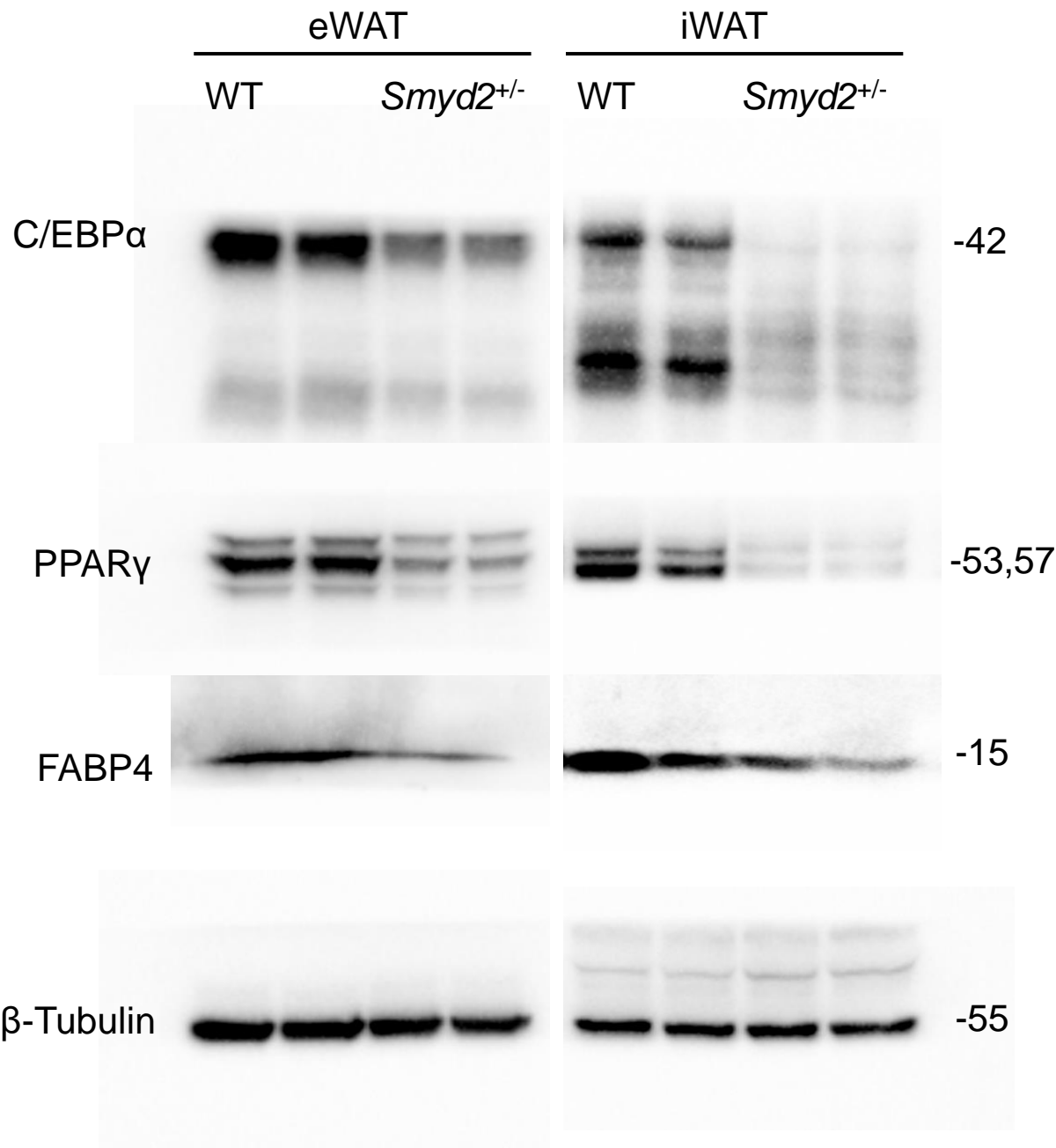

Figure S1

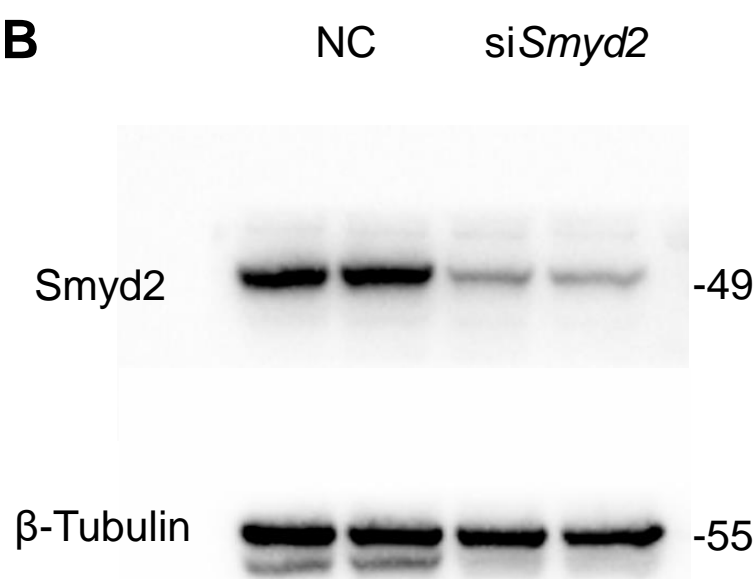

Figure S2

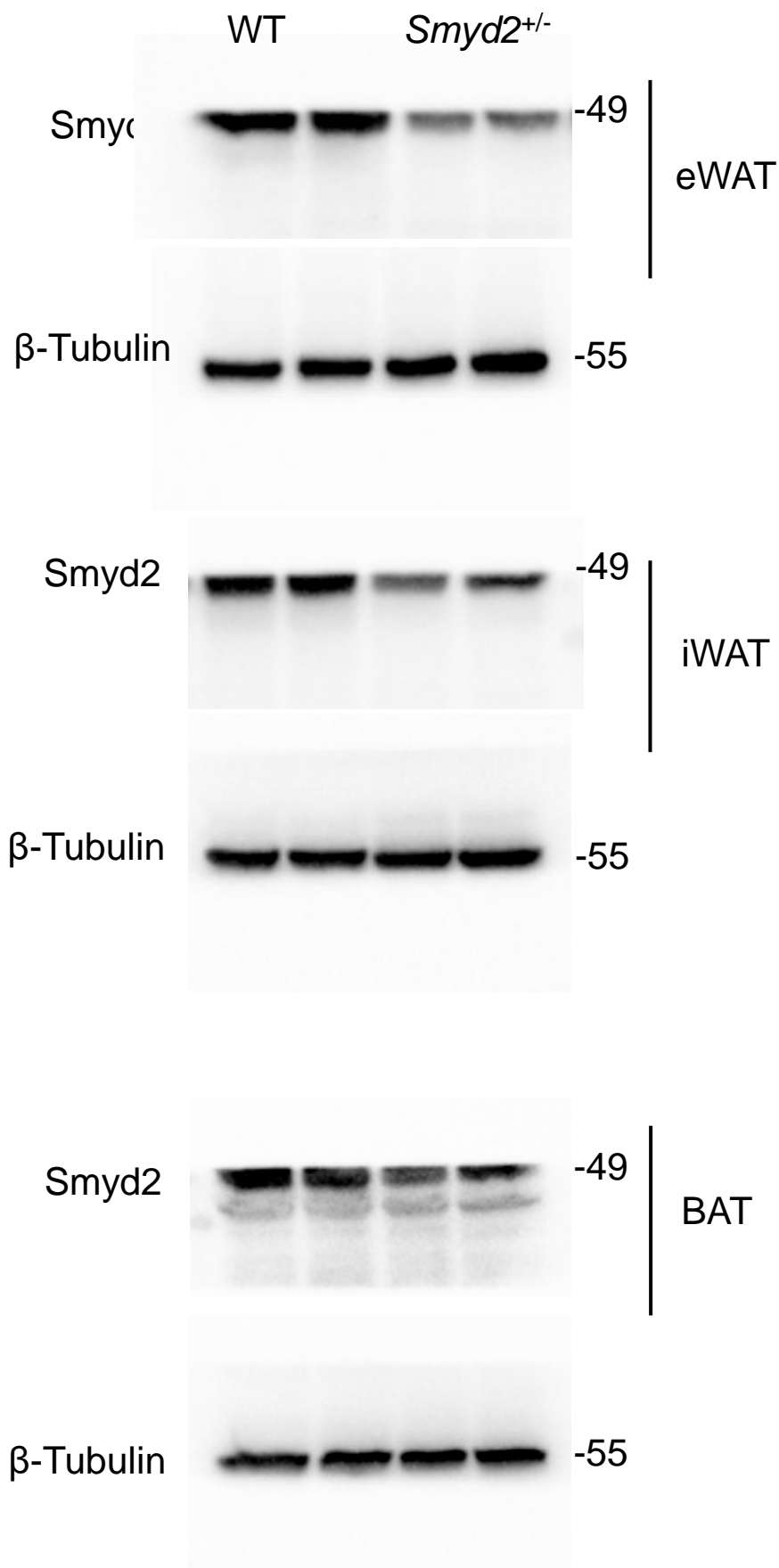

Figure S3

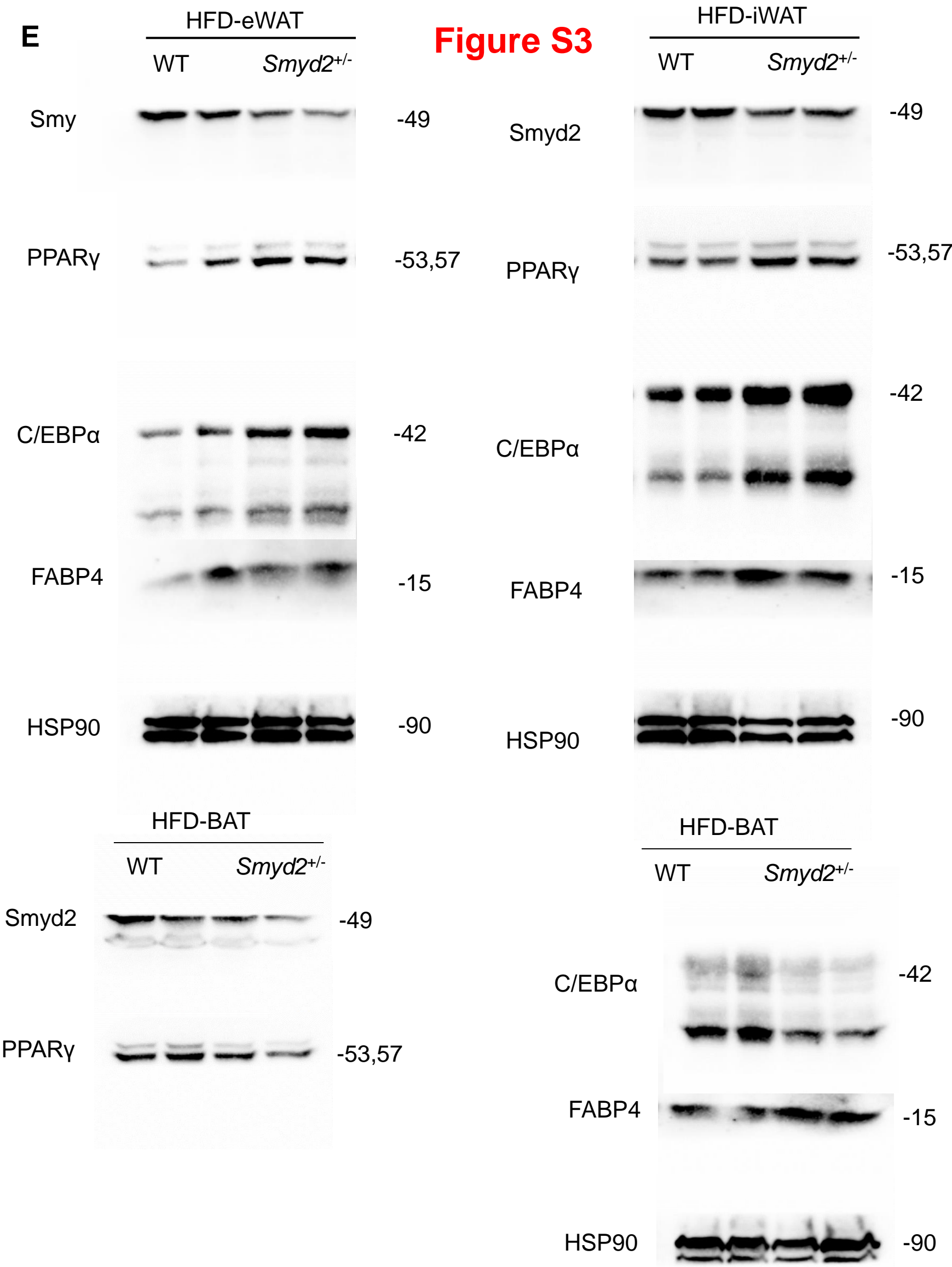

**Figure S4**

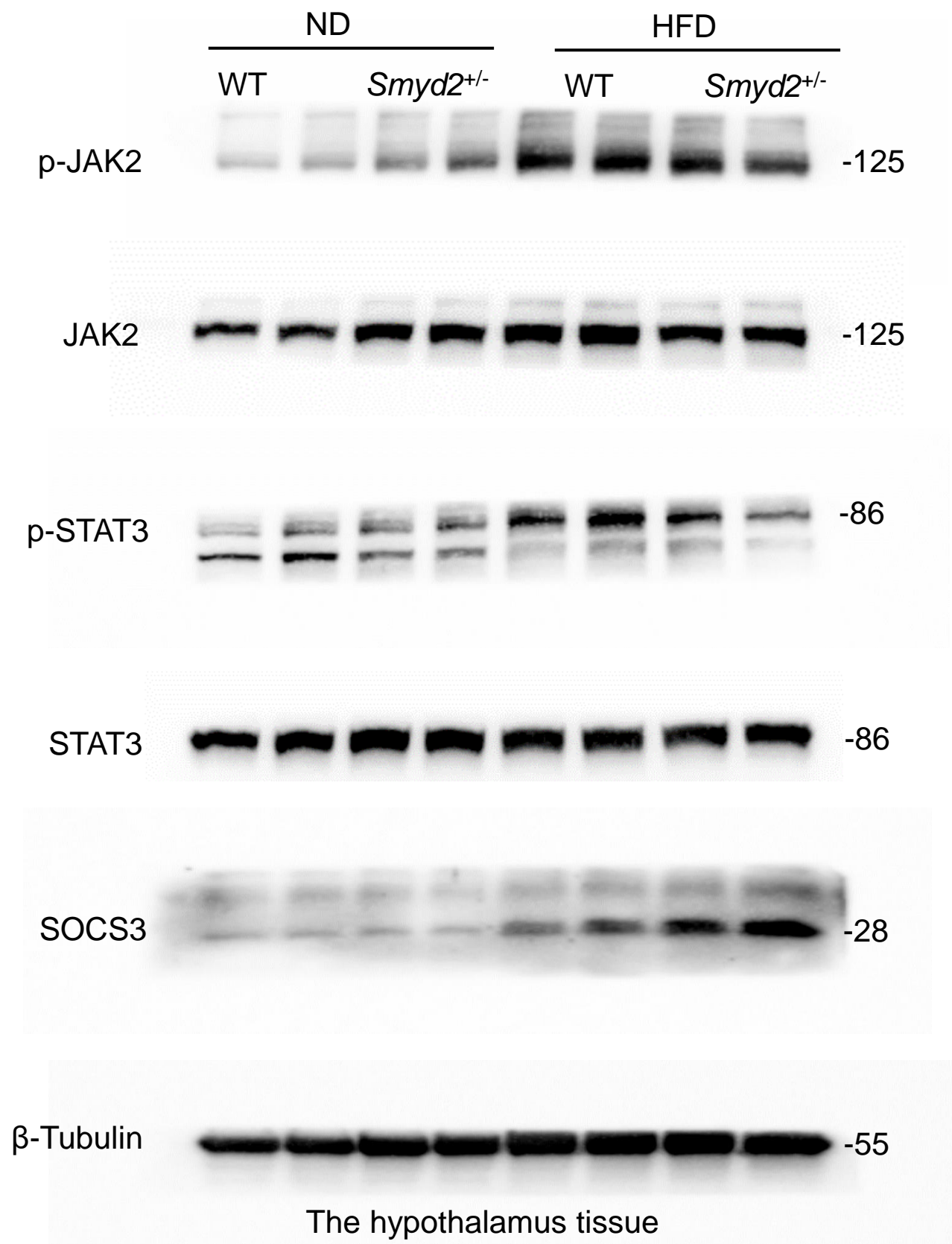

Supplement: Supplementary file 3 — Supplemental Material-WB [file 41419_2022_5321_MOESM3_ESM.pdf]
